# Proteomic and transcriptomic analysis of lung tissue in OVA-challenged mice

**DOI:** 10.1007/s12272-017-0972-4

**Published:** 2017-10-30

**Authors:** Yongjin Lee, Yun-Ho Hwang, Kwang-Jin Kim, Ae-Kyung Park, Man-Jeong Paik, Seong Hwan Kim, Su Ui Lee, Sung-Tae Yee, Young-Jin Son

**Affiliations:** 1 Department of Pharmacy, Sunchon National University, 255 Jungangno, Suncheon, Jeonnam 57922 Korea; 2 Laboratory of Translational Therapeutics, Division of Drug Discovery Research, Pharmacology Research Center, Korea Research Institute of Chemical Technology, Daejeon, 34114 Korea; 3 Natural Medicine Research Center, Korea Research Institute of Bioscience and Biotechnology, Cheongju, Chungcheongbuk 56212 Korea

**Keywords:** Asthma, Ovalbumin (OVA), Environmental respiratory disease, Proteomics, RNA-seq

## Abstract

Asthma is a long term inflammatory disease of the airway of lungs characterized by variable airflow obstruction and bronchospasm. Asthma is caused by a complex combination of environmental and genetic interactions. In this study, we conducted proteomic analysis of samples derived from control and OVA challenged mice for environmental respiratory disease by using 2-D gel electrophoresis. In addition, we explored the genes associated with the environmental substances that cause respiratory disease and conducted RNA-seq by next-generation sequencing. Proteomic analysis revealed 7 up-regulated (keratin KB40, CRP, HSP27, chaperonin containing TCP-1, TCP-10, keratin, and albumin) and 3 down-regulated proteins (PLC-α, PLA2, and precursor ApoA-1). The expression diversity of many genes was found in the lung tissue of OVA challenged moue by RNA-seq. 146 genes were identified as significantly differentially expressed by OVA treatment, and 118 genes of the 146 differentially expressed genes were up-regulated and 28 genes were downregulated. These genes were related to inflammation, mucin production, and airway remodeling. The results presented herein enable diagnosis and the identification of quantitative markers to monitor the progression of environmental respiratory disease using proteomics and genomic approaches.

**Electronic supplementary material:**

The online version of this article (doi:10.1007/s12272-017-0972-4) contains supplementary material, which is available to authorized users.

## Introduction

The prevalence of asthma is increasing worldwide and the complexity and severity of asthma continue to increase in children and adults (Pawankar 2014). Although asthma is effectively treated, the cost of treatment is largely (Barnes et al. 1996). Asthma is a common long term inflammatory disease of the airway of lungs characterized by variable airflow obstruction and bronchial hyperresponsiveness (BHR) (Lemanske and Busse 2003), as well as elevated IgE level and airway hyperreactivity (AHR) (Lapperre et al. 2004). The mechanisms of allergic asthma are related to increased production of T-helper cell (Th)2-cytokines such as IL-4, IL-5, and IL-13 (Lloyd and Hessel 2010) coupled with decreased expression of T-helper cell (Th)1-cytokines such as TNF-α and IFN-γ. Especially, IFN-γ inhibits the proliferation of Th2 cells (Gajewski and Fitch 1988). Therefore, the balance between Th1 and Th2 cells is an important aspect of treatment of allergic asthma (Scherf et al. 2005). The Th2 cytokines, IL-4, IL-5, and IL-13, were increased in patients of allergic asthma. These Th2 cytokines mediate airway eosinophil and mast-cell recruitment, leading to infiltration of inflammatory cells into the lung and development of allergic asthma (Wild et al. 2000; Oh et al. 2010). The Th2 cytokines also induce mucin production (MUC2, MUC5AC, and MUC8), which leads to development of asthma (Koo et al. 2002).

In this study, we applied the proteomic and RNA-seq approach to identify the genes and proteins differentially expressed between control and asthmatic (OVA challenged) lung of mice. Proteomic analysis with two-dimensional polyacrylamide gel electrophoresis (2-DE) is a powerful technique for examining diverse proteomes and analyzing differentially expressed proteins (O’Farrell 1975). RNA-seq of allergic induced mouse lung by next-generation sequencing should facilitate investigation of transcriptomes differentially expressed between normal and asthma (Yick et al. 2013). Therefore, we conducted to investigate genes and proteins differentially expressed and to explore novel markers and to verify the known markers of asthma using a proteomic approach and RNA-seq.

## Materials and methods

### Antibodies and reagents

All commercial antibodies and reagents were purchased from the following resources: anti-phospho-cPLA2 (Ser505) substrate (#2831, 1: 1000 dilution), anti-cPLA2 (#2832, 1: 1000 dilution), anti-phospho-HSP27 (Ser82) (#2401, 1: 1000 dilution), anti-HSP27 (#2402, 1: 1000 dilution), and anti-ApoA1 (#3350, 1: 1000 dilution) antibodies were from Cell Signaling Technology. Ovalbumin (A5503) was purchased from Sigma-Aldrich (St. Louis, USA). Purified rat anti-mouse IgE (R35-72), purified rat anti-mouse IgG1 (A85-3), purified rat anti-mouse IgG2a (R11-89), purified rat anti-mouse IL-4, purified rat anti-mouse IL-5, purified rat anti-mouse IFN-γ, biotin rat anti-mouse IgE (R35-118), biotin rat anti-mouse IgG1 (A85-1), biotin rat anti-mouse IgG2a (19-5), biotin rat anti-mouse IL-4, biotin rat anti-mouse IL-5, and biotin rat anti-mouse IFN-γ were purchased from BD Biosciences (San Diego, USA).

### Animals

Female C57BL/6 mice (6–7 weeks) were bred and maintained under specific pathogen-free conditions at Orientbio (Seongnam, Korea). Animals were housed at a controlled temperature of 22 ± 2 °C and 50 ± 5% relative humidity. Mice were housed in polycarbonate cages and fed a standard diet with water. All mice were treated in strict accordance with Sunchon National University Institutional Animal Care and Use Committee (SCNU IACUC) guidelines for the care and use of laboratory animals. All procedures were approved by the SCNU IACUC (permit number: SCNU IACUC-2015-04). All experiments were performed under sodium pentobarbital anesthesia, and every effort was made to minimize suffering.

### Sensitization and Provocation of Airway Inflammation with OVA

**Fig. 1:**
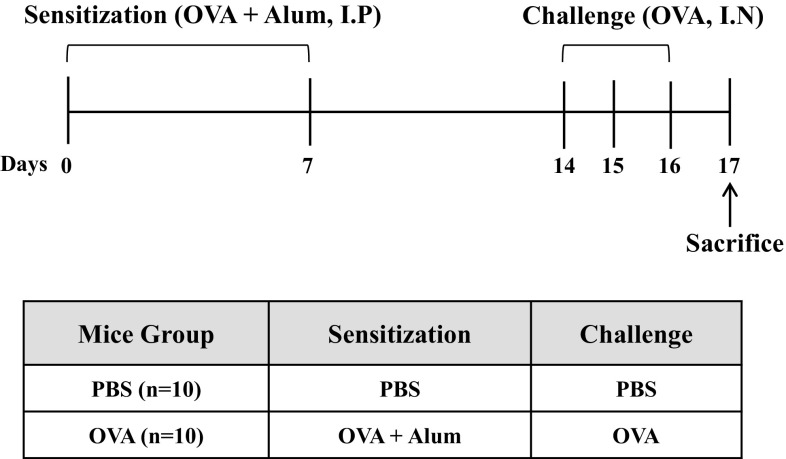
Experimental scheme for induction of airway inflammation in mouse model

Mice were randomly divided into two groups of ten animals (10 mice per group). C57BL/6 mice were primary sensitized by the intraperitoneal injection of 10 mg/mL of OVA or vehicle in 0.2 mL of saline on day 0 and 7. The mice were challenged with PBS or 10 μg of OVA dissolved in 50 μL of saline (intranasal injection) under anesthesia on days 14, 15, and 16 (Fig. 1). 24 h after the last airway challenge, blood was collected in a retro orbital plexus. After centrifugation (3500×*g*, 4 °C, 5 min), the serum was stored at − 20 °C until assayed for immunoglobulins by ELISA. The animals were sacrificed by cervical dislocation. Bronchoalveolar lavage (BAL) of the mice was performed four times each with 0.5 mL of saline. After centrifugation (500×*g*, 4 °C, 5 min), the supernatant of BAL obtained from 2 mL of instilled saline was stored at − 20 °C until assayed for cytokines by ELISA. The red blood cells in BAL were removed by tris-buffered ammonium chloride. The total cells were counted using a hemacytometer.

### Assessment of airway hyperresponsiveness in OVA-induced asthmatic mice

The commercial brand of the Aeroneb used should be indicated as well as the mean diameter of produced drops. This is important to understand the level of action of metacholine in the bronchial tree. After final challenge, mice were weighed and anesthetized by injection of Zoletil and Rumpun. The mice were then tracheostomized using an 18G metal cannula, after which they were placed in a flow-type body plethysmograph and connected to a small-animal ventilator by the endotracheal cannula (FlexiVent, SCIREQ Inc., Montreal, Canada). Doses of methacholine (MCh) were administered using a nebulizer (Aeroneb) and progressively doubling concentrations ranging from 12.5 to 50 mg/mL. Respiratory system resistance (Rrs) and respiratory system elastance (Ers) were determined before challenge and after each dose of MCh. The parameters tissue damping (G) and tissue dynamic elastance (H) were computed by multiple linear regression.

Aeroneb (FINE MIST ANP-1100) specifications: particle size (VMD-Volume Median Diameter; 3.5 μm), nominal nebulization output rate (> 0.1 mL/min), residual volume (< 0.2 mL), mass median aerodynamic diameter (MMAD; ~ 1.8 μm), geometric standard deviation (GSD; 2.0 μm).

### Measurement of inflammatory cytokine and immunoglobulin production in OVA-induced asthmatic mice

Levels of various cytokines such as IL-4, IL-5, and IFN-γ in BAL and immunoglobulins (Igs) such as total IgE, OVA-specific IgG1, and IgG2a in serum were measured by enzyme-linked immunosorbent assay (ELISA).

### Histological examination of mouse lung tissue

After BAL isolation, the mouse left lungs were fixed in 10% formalin. Four-micron sections were cut and stained with Hematoxylin & Eosin (H&E) (MERCK, Kenilworth, USA) and Alcian blue-PAS (Sigma, St. Louis, USA) and stained with antibody against caspase-1 (1:200; Abcam, Cambridge, UK).

### Sample preparation

Fresh lung tissue from control and OVA-treated mice were placed in a clean mortar containing liquid nitrogen and pulverized. Samples were sonicated and purified with lysis buffer containing 7 M urea, 2 M thiourea, 4% CHAPS, 2.5% DTT, and protease inhibitor. The lysate was centrifuged at 12,000×*g* for 30 min, after which supernatants of each group were pooled and transferred to clean vials. The protein concentrations were determined using a BCA assay kit (Pierce, Waltham, USA) and stored at − 70 °C until used.

### Sample preparation and 2-DE

Sample preparation and 2-DE were performed as described by (Bahk et al. 2004). The lysates were then homogenized and centrifuged at 15,000×*g* for 20 min, after which they were suitably stored at − 80 °C. Protein concentration was determined by the Bradford method (BioRad, Hercules, USA). For 2-DE analysis, pH 4–7 IPG strips (GE Healthcare Life Sciences, Pittsburgh, USA) were rehydrated in swelling buffer containing 7 M urea, 2 M thiourea, 2.5% (w/v) DTT, and 4% (w/v) CHAPS. The gels were destained using deionized water and images were acquired with an image scanner (BioRad, Hercules, USA). Image analysis was carried out using ImageMaster™ 2D Platinum software (Amersham Biosciences, Hercules, USA). For comparison of protein spots, more than 25 spots in all gels were correspondingly landmarked and normalized.

### In-gel digestion with trypsin and extraction of peptides

In-gel digestion of protein spots from Coomassie Blue stained gels was carried out as previously described (Bahk et al. 2004). Briefly, selected protein spots were excised from stained gels and cut into pieces. The gel pieces were washed for 1 h at room temperature in 25 mM ammonium bicarbonate buffer, pH 7.8, containing 50% (v/v) acetonitrile (ACN). Following the dehydration of gel pieces in a centrifugal vacuum concentrator for 10 min, gel pieces were rehydrated in 50 ng of sequencing grade trypsin solution (Promega, Madison, USA). After incubation in 25 mM ammonium bicarbonate buffer, pH 7.8, at 37 °C overnight, the tryptic peptides were extracted with 5 µL of 0.5% formic acid including 50% (v/v) ACN for 40 min with mild sonication. The extracted solution was concentrated using a centrifugal vacuum concentrator. Before mass spectrometric analysis, the peptides solution was conducted to a desalting process using a reversed-phase column (Gobom et al. 1999). Briefly, after an equilibration step with 10 µL of 5% (v/v) formic acid, the peptides solution was loaded onto the column and washed with 10 µL of 5% (v/v) formic acid. The bound peptides were eluted with 5 µL of 70% ACN with 5% (v/v) formic acid.

### Identification of proteins by LC–MS/MS

After desalting, eluted tryptic peptides were separated and analyzed using a nano ACQUITY UPLC (Waters, Milford, USA) directly coupled to a Finnigan LCQ DECA iontrap mass spectrometer (Thermo Scientific, Waltham, USA). Briefly, the peptides were bound to the ACQUITY UPLC peptide BEH C18 column (1.7 µm, 130 Å pore size, 100 µm × 100 mm) with distilled water containing 0.1% (v/v) formic acid and the bound peptides were eluted with a 40 min gradient of 0–90% (v/v) acetonitrile containing 0.1% (v/v) formic acid at a flow rate of 0.4 µL/min. For tandem mass spectrometry, the full mass scan range mode was m/z = 400–2000 Da. After determination of the charge states of an ion on zoom scans, product ion spectra were acquired in MS/MS mode with a relative collision energy of 55%. We set the modifications of methionine and cysteine for MS analysis. The MS/MS ion mass tolerance was 1 Da, allowance of missed cleavage was 1, and charge states (+ 1, + 2, and + 3) were taken into account for data analysis. We took only significant hits as defined by MASCOT probability analysis.

### RNA-seq analysis of OVA-induced asthmatic mice

The libraries were prepared for 100 bp paired-end sequencing using a TruSeq RNA Sample Preparation Kit (Illumina, San Diego, USA). Namely, mRNA molecules were purified and fragmented from 2 μg of total RNA using oligo (dT) magnetic beads. The fragmented mRNAs were synthesized as single-stranded cDNAs through random hexamer priming. By applying this as a template for second strand synthesis, double-stranded cDNA was prepared. After end repair, A-tailing and adapter ligation, cDNA libraries were amplified by PCR (Polymerase Chain Reaction). The quality of these cDNA libraries was evaluated using the Agilent 2100 BioAnalyzer (Agilent, Santa Clara, USA). Samples were quantified with the KAPA library quantification kit (Kapa Biosystems, Wilmington, USA) according to the manufacturer’s protocols. Following cluster amplification of denatured templates, paired end (2 × 100 bp) sequencing was conducted using an Illumina HiSeq 2500 (Illumina, San Diego, USA).

For DEG analysis, gene level count data were generated using HTSeq-count v0.6.1p1 with the option “-m intersection-nonempty” and “-r option considering paired-end sequence.” Based on the calculated read count data, DEGs were identified using the TCC R package (Sun et al. 2013), which applies robust normalization strategies to compare tag count data. Normalization factors were calculated using the iterative DEGES/edgeR method. The Q-value was calculated based on the *p* value using the p. adjust function of the R package with the default parameter settings. The DEGs were identified based on a q-value threshold less than 0.05 for correcting errors caused by multiple-testing.

### Real-time quantitative RT-PCR (qRT-PCR)

Total RNA was extracted from frozen lung tissue samples using the TRIzol Reagent (Thermo Fisher, Waltham, USA). Complementary DNA was synthesized from total RNA using an M-MLV cDNA synthesis kit (Enzynomics) and oligo (dT) primer. Real-time polymerase chain reaction (PCR) analysis was performed using SYBR green dye to measure duplex DNA formation with the BioRad Real-Time PCR Detection System and normalized to the expression of GAPDH. The following primers and probes were used: IL-4 sense 5′-GGTCTCAACCCCCAGCTAGT-3′, antisense 5′-GCCGATGATCTCTCTCAAGTGAT-3′; IL-13 sense 5′-CCTGGCTCTTGCTTGCCTT-3′, antisense 5′-GGTCTTGTG TGATGTTGCTCA-3′; PLA2 sense 5′-AGCAGGCAGAGCGATATGAT-3′, antisense 5′-TTCTCAGCACCTTCCGTCTT-3; ApoA1 sense 5′-GTGGCTCTGGTCTTCCTGAC-3′, antisense 5′-ACGGTTGAACCCAGAGTGTC-3′; CRP sense 5′-CCAGC ATATGGGCATACCTT-3′, antisense 5′-CAGACCTCAGTGGCTCCTTC-3′; HSP27 sense 5′-CCTCTTCGATCAAGCTTTCG-3′, antisense 5′-GCCTTCCTTGGTCTTCACTG-3′; GAPDH sense 5′-GACCCCTTCATTGACCTC-3′, antisense 5′-GCCATCCAC AGTCTTCTG-3′.

### Western blotting

Lung tissues were placed on ice and extracted with lysis buffer containing 20 mM Tris–HCl, pH 7.5, 1% v/v Igepal CA-630, 150 mM NaCl, 1 mM sodium fluoride (Sigma, St. Louis, USA), 1 mM sodium orthovanadate (Sigma, St. Louis, USA), 0.5 mM phenylmethylsulfonyl fluoride (Sigma, St. Louis, USA), 1 mM DTT, 2 mM EDTA, 10 μg/mL Aprotinin (Sigma, St. Louis, USA), 5 µg/mL Leupeptin (Sigma, St. Louis, USA), and 2 µg/mL Pepstatin (Sigma, St. Louis, USA). Lysates were centrifuged at 12,000×*g* for 15 min at 4 °C. Protein extracts were loaded onto SDS-PAGE and transferred to PVDF membranes (Millipore, Billerica, USA). The membranes were blocked for 1 h in TTBS (Tris-buffered saline containing 0.1% Tween 20) containing 5% skim milk, and immunoblotting was conducted by overnight incubation, followed by incubation with secondary antibodies conjugated to horseradish peroxides (HRP) for 3 h at room temperature. After washing, the bands-of interests were viewed using a luminescent image analyzer MicroChemi 4.2 (DNR Bio-Imaging System, Jerusalem, Israel).

### Statistical analysis

The proteins and RNA from the asthmatic and control groups were analyzed for differences in levels of expression using a Student’s t-test or the Mann–Whitney U-test. Values of *p* < 0.05 were considered significant.

## Results

### Assessment of airway responsiveness to methacholine

**Fig. 2:**
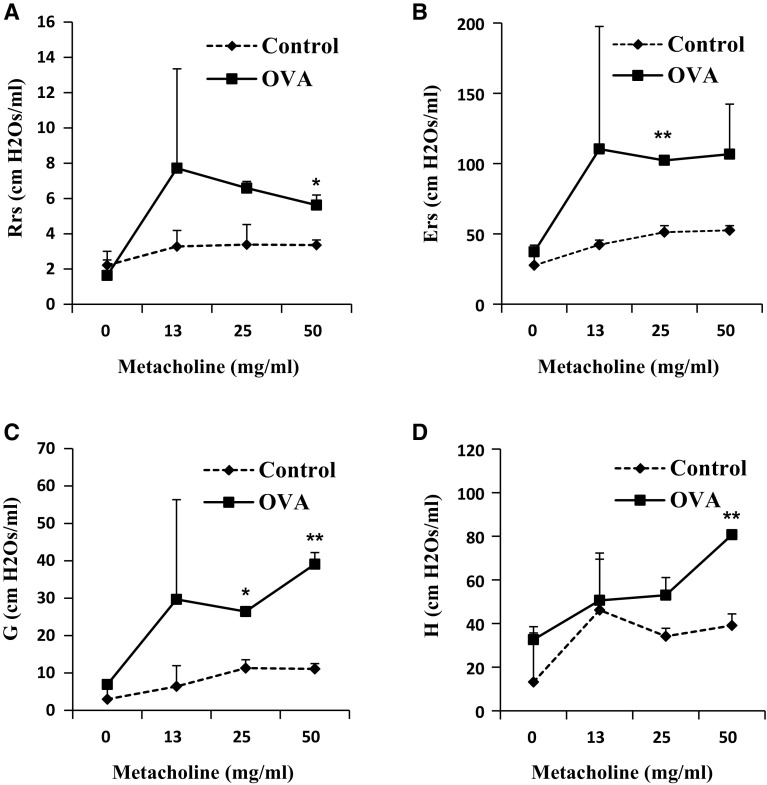
Assessment of OVA-induced airway hyperresponsiveness by the forced oscillation technique. **a** Respiratory system resistance (Rrs), **b** elastance (Ers), **c** tissue damping (G), and **d** tissue elastance (H) were determined using an OVA-induced asthma mouse model. *p < 0.05, and **p < 0.01 compared with control

The OVA group exhibited higher airway responsiveness to MCh than the control group. The Rrs and the Ers were significantly higher in the OVA group, being 50 and 25 mg/mL of MCh, respectively (Fig. 2a, b). Moreover, the OVA group exhibited a significant higher response than the control group in the parameters reflecting the peripheral tissues, such as G corresponding to tissue damping and H to the Dynamic Elastance (Fig. 2c, d).

### Assessment of cytokines, immunoglobulins, and BAL total cells in OVA treated with mice

**Fig. 3:**
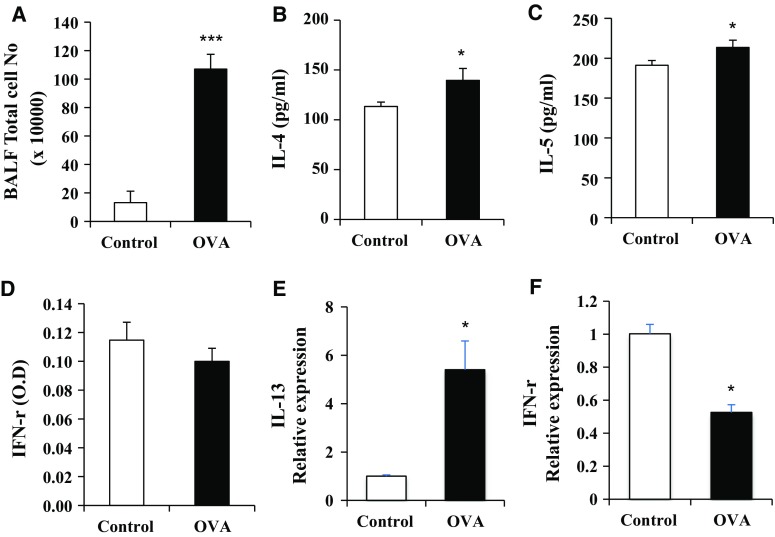
BALF total and inflammatory cytokines levels in mice exposed to OVA. Asthma was induced as described in the Materials and Methods. BALF was collected 24 h after the last challenge and (**a**) total cells were counted. **b** IL-4, **c** IL-5, and **d** IFN-γ were measured by ELISA. IL-13 (**e**) and IFN-r (**f**) mRNA expression in OVA-treated lung tissues are shown *p < 0.05, and ***p < 0.001 compared with control

**Fig. 4:**
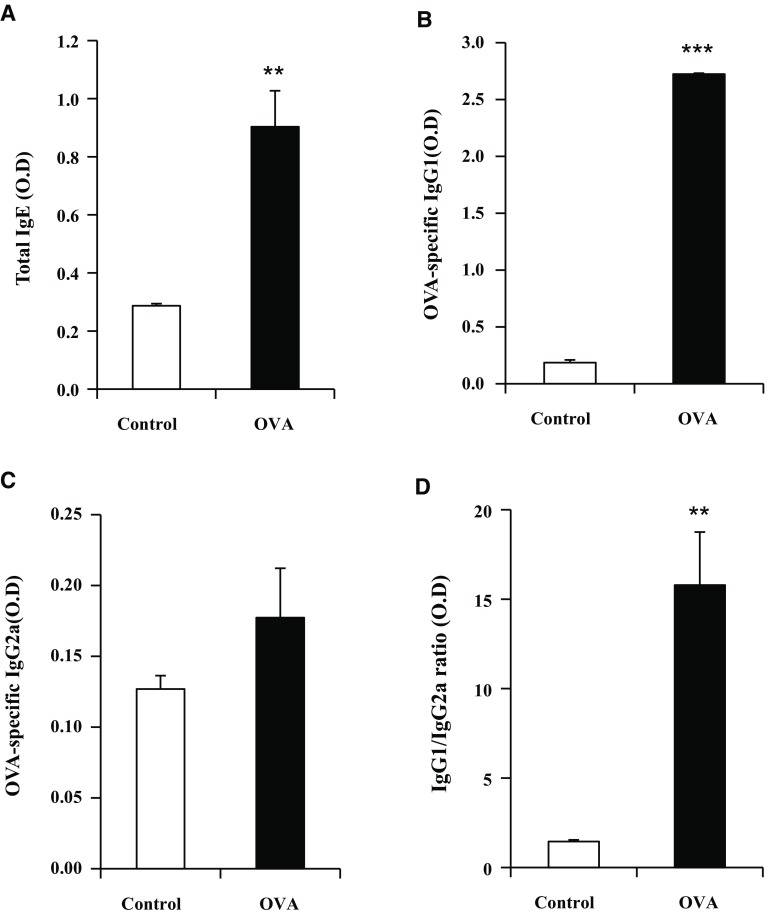
Levels of immunoglobulins in mice exposed to OVA. Asthma was induced as described in the Materials and Methods. **a** Total IgE, **b** OVA-specific IgG1, **c** OVA-specific IgG2a, and **d** IgG1/IgG2a ratio were measured by ELISA. **p < 0.01, and ***p < 0.001 compared with control

T-helper type 2 (Th2) cells are involved in allergic airway inflammation and mainly produce IL-4, IL-5, and IL-13 (Fernandes et al. 2003). Conversely, IFN-γ secreted from Th1 cells protects against allergic disease by suppressing the activity of Th2 cells. Moreover, IgG1 and IgG2a immunoglobulin isotypes are markers for Th2 and Th1 lymphocytes, respectively (Mountford et al. 1994; Hansen et al. 1999). In this study, the total cells in BALF, IL-4, and IL-5 increased in the OVA group relative to the control group (Fig. 3a, b, c). IFN-γ was not significantly decreased in the OVA group relative to the control group (Fig. 3d). The mRNA expression of IL-13 and IFN-γ was measured by real-time qPCR (Fig. 3e, f). IL-13 was significantly elevated, and IFN-γ was much lower in the OVA group than the control group. The total IgE and OVA-specific IgG1 in the OVA group were significantly increased when compared with the control group (Fig. 4a, b). However, the OVA-specific IgG2a levels did not differ significantly between the control and OVA group (Fig. 4c). OVA-specific IgG1/IgG2a was significantly increased in the OVA group relative to the control group (Fig. 4d).

### The adverse effects of OVA on lung tissue of mice

**Fig. 5:**
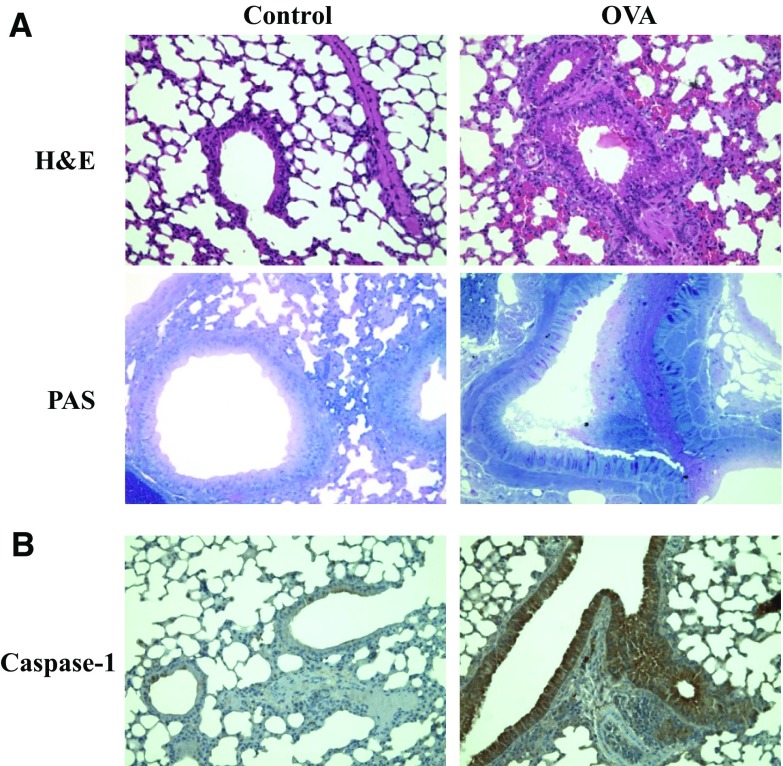
The histopathological changes in the lung tissue of OVA challenged mice. The lung tissues of OVA challenged mice stained with **a** H&E (×200) and PAS (×400). **b** Immunohistochemisty of caspase-1 (×200) in the lung. Data represent fine mice per group

H&E staining indicates that peribronchial and perivascular infiltrations of lymphocytes and eosinophils were significantly worse in the OVA group compared with the control group. Further, PAS stained goblet cell of bronchial mucosal secretions was higher in the OVA group compared with the control group (Fig. 5a). The other evidence of airway inflammation was assessed by caspase-1 immunohistochemistry. Caspase-1 expression was increased in OVA group compared with control group (Fig. 5b).

### 2-DE analysis of control mice, OVA-treated mice

In Figs. 2, 3 and 4, asthmatic markers induced by OVA were measured and the most effective asthma-induced mice were selected one by one in each group. Lungs were isolated from control and OVA-treated mice. Proteins from individual mice were combined in each experimental group. The protein lysates (700 µg) were loaded into the rehydrated IPG strips using an IPGphor III (GE Healthcare Life Sciences, Pittsburgh, USA), after which 2-D separation was performed on 12% sodium dodecyl sulfate–polyacrylamide gels (SDS-PAGE). Following fixation of the gels for 1 h in a solution of 40% (v/v) methanol containing 5% (v/v) phosphoric acid, the gels were stained with Colloidal Coomassie Blue G-250 solution (ProteomeTech, Seoul, Korea) (Fig. 5). 2-D gel patterns were then analyzed using the ImageMaster™ 2D Platinum software. For comparison of protein spots, more than 25 spots in all gels were correspondingly landmarked and normalized.

### Differential protein expression and protein identification

**Fig. 6:**
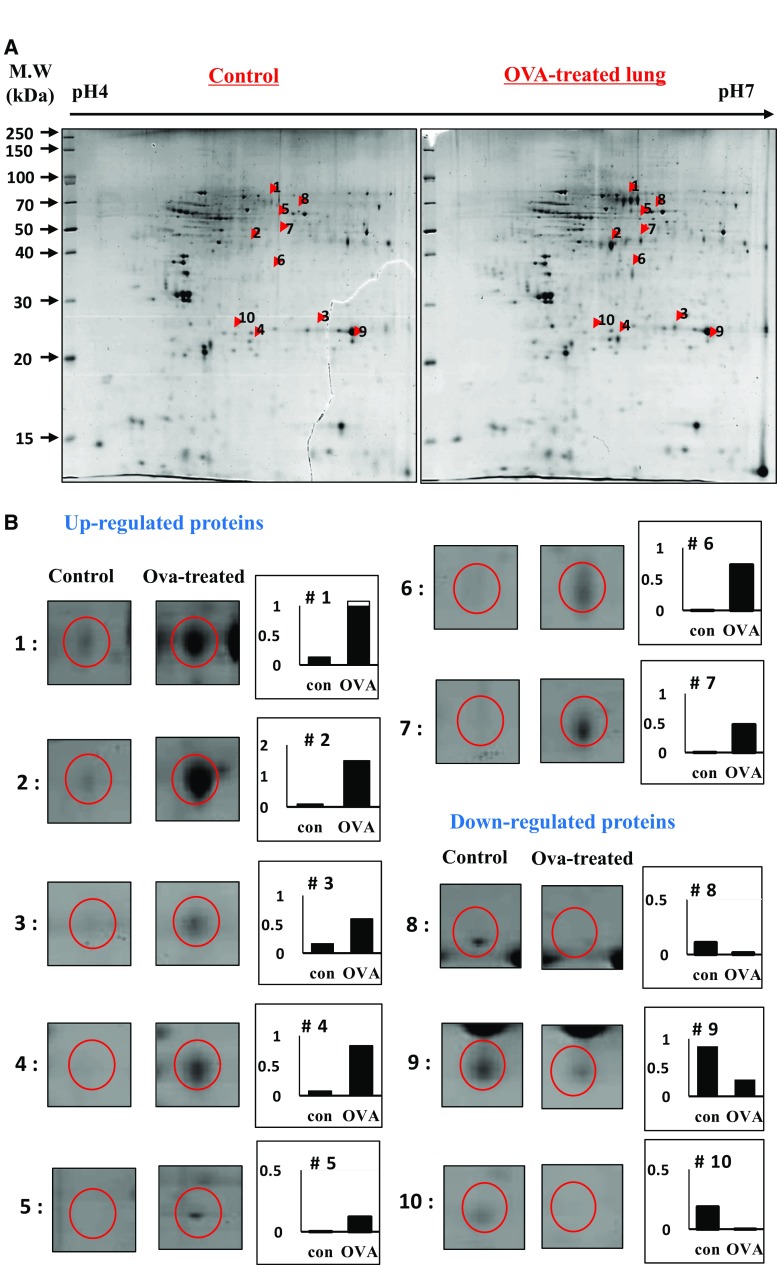
2-DE analysis of OVA-induced allergic asthma in mouse lung tissues. 2-dimention electrophoresis (DE) of proteins from control and OVA-treated were preformed (**a**) and ten differentially expressed proteins are marked by their spot numbers in the control and OVA-treated 2-DE gels (**b**). These proteins were identified by MALDI TOF/TOF, and the proteins they encode are listed in Table 1

Many spots were found to be differentially expressed between normal and OVA-treated mice upon 2DE analysis (Fig. 6a). Of these, ten proteins that showed great differential expression were selected for further analysis, seven that increased and three that decreased (Fig. 6b).

The individual spectra from MS/MS were processed using the SEQUEST software (Thermo Quest, San Jose, USA), and the generated peak lists were used to query the NCBI database with the MASCOT program (Matrix Science Ltd., London, UK). Up-regulated proteins in OVA-treated tissues contained keratin KB40, chitinase-related protein (CRP), heat shock protein HSP27, chaperonin containing TCP-1, TCP-10, keratin, and albumin. Down-regulated proteins showed phospholipase C-α (PLC-α), phospholipase A2 (PLA2), and precursor apolipoprotein A-1 (ApoA1) (Table 1).

**Table 1:** Differentially expressed proteins in the lungs of mice with OVA-induced allergic asthma

Spot no.	Protein name	Protein accession no.	Change in intensity Con/OVA	Theoretical MW(kDa)/pI	MOWSE score	Percent sequence coverage (%)
Up-regulated protiens in allergic asthuma						
1	Keratin KB40	gi 46485130	8.02	17/5.20	35	9
2	Chitinase-related protein MCRP	gi 1336166	16.09	29/5.14	151	13
3	Heat shock protein HSP27	gi 424143	3.63	23/6.45	66	15
4	Chaperonin containing TCP-1	gi 468546	10.59	57/5.97	48	3
5	Keratin, type II cytoskeletal 7	gi 14861854	85.16	50/5.67	149	17
6	Chaperonin containing TCP-10	gi 53994	549.05	49/5.94	36	7
7	Albumin	gi 148693135	292.31	69/5.75	391	13
Up-regulated protiens in allergic asthuma						
8	Phospholipase C-α	gi 200397	6.71	56/5.98	68	2
9	Phospholipase A2	gi 3219774	2.98	25/5.71	145	25
10	Precursor Apolipoprotein A-I	gi 160333304	198.37	30/5.51	207	29

**Fig. 7:**
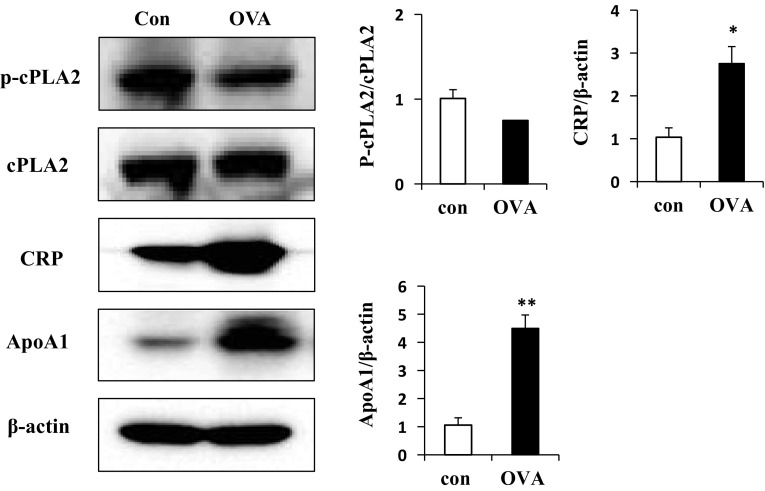
Profiles of OVA-related proteins in OVA-induced lung tissues. Tissue proteins were extracted from OVA-induced mice and used to assay the p-cPLA2, CRP and ApoA1 by Western blot. *p < 0.05 and **p < 0.01, compared with control

**Fig. 8:**
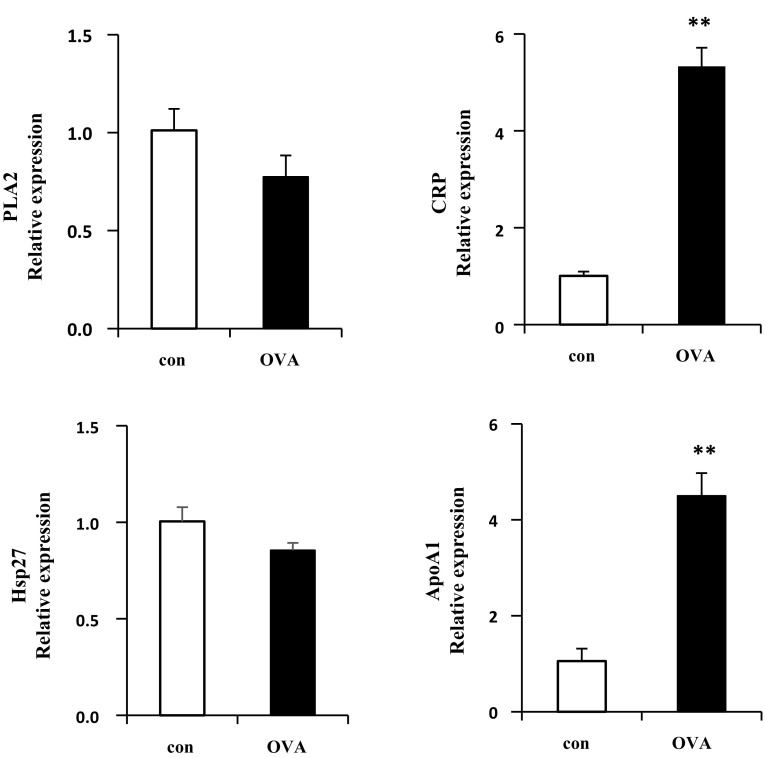
mRNA expression level of OVA-induced lung tissue. The mRNA expression of HSP27, ApoA1, cPLA2 and CRP were up- and down-regulated by OVA treated mice (n = 10). **p < 0.01, compared with control

To verify our 2-D electrophoresis results, we conducted real-time qPCR and western blotting. The expression of CRP and ApoA1 were significantly increased, but the expression of phospholipase A2 was decreased compared with control mice (Fig. 7). The mRNA expression level of CRP and ApoA1 in OVA-treated mice were significantly increased but PLA2 was decreased compared with control mice. Interestingly, ApoA1 was inconsistent with Proteomic data, in other words, the levels of mRNA and protein of ApoA1 were significantly increased in OVA challenged mice compared with Proteomics data (Fig. 8).

### Regulation of asthmatic genes by OVA treatment in mouse lung tissues

The mRNA sequencing was performed using TruSeq RNA Library Preparation Kit and TruSeq RNA Access Kit (Illumina, San Diego, USA). Whole-transcriptome sequencing was performed using an Illumina HiSeq 2500 platform. We used in-house script to trim low quality reads (N-ratio > 0.1 and ratio of less than Phred quality score 20 > 0.4) and then filtered sequence reads were mapped onto the mouse reference genome (Ensembl v77). We used htseq and the R package TCC to calculate differentially expressed genes, and observed the numbers of potential DEGs satisfying FDR < 0.05 in the DEG data set. The candidate genes were performed with GeneOntology analysis and then identified from GO ter m (Table S1).

**Fig. 9:**
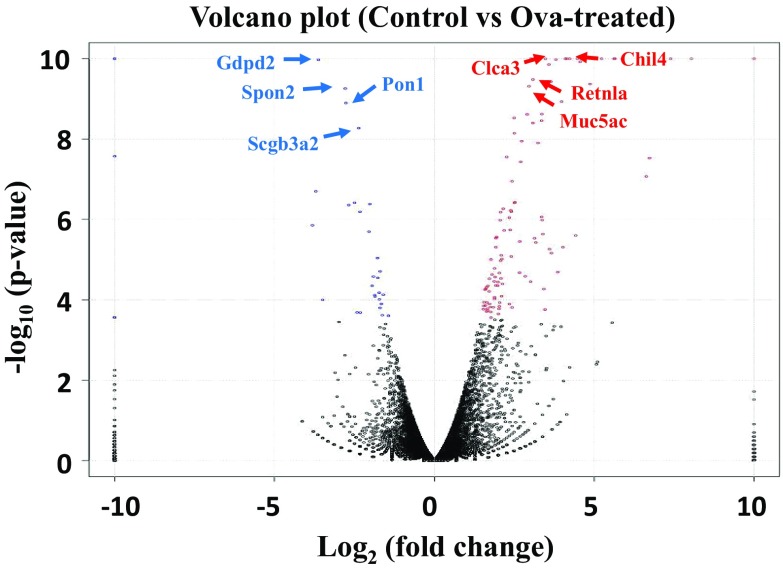
Volcano-plot of genes of asthmatic (OVA-challenged) mice versus control. The logarithms of the fold changes of individual genes (x-axis) are plotted against the negative differentially expressed genes between asthma and controls with p < 0.05 after correction for multiple testing (Cutoff: q-value < 0.05)

To investigate the differential expression of RNA in the lung tissues of OVA-induced mice, we conducted RNA-seq for transcriptome. As a result of the RNA-seq performed in this study, 1 ILLUMINA run (Illumina HiSeq 2500) was performed in control samples to obtain information on RNAs of 20,232,248 spots and 4.1 G bases. In OVA-treated samples, 24,676,340 spots and 5 G bases. Many genes were expressed differentially between control and OVA-treated lung tissues. In this study, total number of genes obtained by RNA-seq analysis was 41,393 and the number of transcripts was 100,249. Among them, 20,461 genes were involved in the control and 20,932 genes were involved in OVA-treated mice. The transcriptomes mapped to 10,804 unique genes for upregulated and 10,627 for downregulated genes of OVA-challenged mice (Fig. 9). With extremely stringent conditions (q-value < 0.05), 146 genes were selected by filtration and identified as significantly differentially expressed by OVA treatment. We divided differentially expressed genes into those that were up-regulated and down-regulated. The 118 genes of the 146 differentially expressed genes were up-regulated and 28 genes were downregulated (Table S2). Moreover, the gene ontology (GO) terms were used to classify genes into three categories with stringent cutoff (p-value < 0.001), those involved in molecular function (24 genes), biological processes (159 genes), and cellular components (5 genes) (Table S3). We showed significantly high level 20 genes among up-regulated genes and 20 genes of down-regulated gene in Table 2. Some of these genes were involved in inflammation, mucin production, and airway remodeling.

**Table 2:** List of top differentially expressed genes in OVA-challenged and control mice (q-value < 0.05)

Name	Description	Control	OVA	Log_2_ (^OVA^/_con_)	Chromo-some
Up-regulated genes					
Chil4	Chitinase-like 4	7.9	14446.8	10.8	3
Clca3	Chloride channel calcium activated 3	451.1	75920.4	7.4	3
Retnla	Resistin like alpha	1333.1	78933.6	5.9	16
Muc5ac	Mucin 5, subtypes A and C	106.9	5313.6	5.6	7
Slc26a4	Solute carrier family 26, member 4	178.9	5677.2	5	12
Mmp12	Matrix metallopeptidase 12	70.9	2652.3	5.2	9
Ccl24	Chemokine (C–C motif) ligand 24	16.9	829.8	5.6	5
Saa3	Serum amyloid A 3	70.9	1328.4	4.2	7
Scin	Scinderin	28.1	680.4	4.6	12
Cxcl10	Chemokine (C-X-C motif) ligand 10	31.5	700.2	4.5	5
Agr2	Anterior gradient 2	34.9	597.6	4.1	12
Ccl8	Chemokine (C–C motif) ligand 8	13.5	317.7	4.6	11
Cxcl9	Chemokine (C-X-C motif) ligand 9	7.9	229.5	4.9	5
Fbp1	Fructose bisphosphatase 1	1.1	295.2	8	13
Fgf23	Fibroblast growth factor 23	0	45.9	5.4	6
Rnase2a	Ribonuclease, RNase A family, 2A	0	530.1	8.9	14
Capn9	Calpain 9	0	260.1	7.9	8
Fcgbp	Fc fragment of IgG binding protein	229.5	5583.6	4.6	7
Fer1l6	fer-1-like 6 (C. elegans)	94.5	2422.8	4.7	15
Sprr2a3	Small proline-rich protein 2A3	0	105.3	6.6	3
Down-regulated genes					
Gdpd2	Glycerophosphodiester phosphodiesterase domain containing 2	640.12	52.2	−3.6	X
Spon2	Spondin 2, extracellular matrix protein	1593	232.2	−2.8	5
Pon1	Paraoxonase 1	1292.62	190.8	−2.8	6
Scgb3a2	Secretoglobin, family 3A, member 2	17451	3397.5	−2.4	18
Ighv2-3	Immunoglobulin kappa chain variable 2-3	199.12	15.3	−3.7	12
Sult1d1	Sulfotransferase family 1D, member 1	625.5	111.6	−2.5	5
Cyp2f2	Cytochrome P450, family 2, subfamily f, polypeptide 2	66559.5	16542	−2.0	7
Npas2	Neuronal PAS domain protein 2	445.5	70.2	−2.7	1
Arntl	Aryl hydrocarbon receptor nuclear translocator-like	817.88	163.8	−2.3	7
FMO3	Flavin containing monooxygenase 3	1609	392	−2	1
Eln	Elastin	10013	2945	−1.8	5
Bpifa1	BPI fold containing family A, member 1	13106	4056	−1.7	2
Hmgcs2	3-Hydroxy-3-methylglutaryl-Coenzyme A synthase 2	862	230	−1.9	3
Aldh3a1	Aldehyde dehydrogenase family 3, subfamily A1	618	161	−1.9	11
Klf15	Kruppel-like factor 15	1296	394	−1.7	6
Myh6	Myosin, heavy polypeptide 6	5235	1742	−1.6	14
Pdk4	Pyruvate dehydrogenase kinase, isoenzyme 4	648	179	−1.9	6
Dcdc2a	Doublecortin domain containing 2a	631	175	−1.9	13
Acot1	Acyl-CoA thioesterase 11	1035	316	−1.7	12
Asgr1	Asialoglycoprotein receptor 1	101	9	−3.5	11

## Discussion

Asthma and chronic obstructive pulmonary disease (COPD) are common long term inflammatory diseases of the airways of the lungs (Nagano et al. 2014). To understand how asthmatic (OVA-induced) inflammation regulates respiratory disease and how to engage in which genes and proteins cause respiratory diseases, we compared differences between normal and asthmatic lung tissues from mice by proteomic analysis and the RNA-seq method.

Analysis by 2-D electrophoresis revealed 31 proteins differentially expressed in control- and OVA-treated lung tissues of mice, and 25 proteins were up-regulated and 6 proteins were down-regulated in response to OVA. Of these, 10 spots showing large differences were excised from stained gels, cut into pieces, trypsinized, and analyzed by LC–MS/MS. The proteins identified included asthmatic proteins (CRP, HSP27, PLA2, PLC-α, and ApoA-1) and cytoskeletal proteins (Keratin KB40, cytoskeletal 7, chaperonin containing TCP-1, and TCP-10). Of these, keratin KB40, CRP, heat shock protein HSP27, chaperonin containing TCP-1, TCP-10, keratin, and albumin were up-regulated and PLC-α, PLA2, and precursor ApoA-1 were down-regulated.

The proteins CRP, HSP27, PLA2, PLC-α, and ApoA-1 are involved in inflammation. Chitinase-related proteins (CRPs) are a family of mediators increasingly related to infection, T cell-mediated inflammation, wound healing, allergy and asthma. Both chitinases and CLPs are up-regulated under T-helper type 2 (Th2)-driven conditions (Sutherland et al. 2009; Liu et al. 2013). HSP27 is involved in asthma via the p38/HSP27 signaling pathway. p38 MAPK activation by pollutants or stimuli leads to the phosphorylation of HSP27, which results in thickening of the airway wall and obstruction of airflow (Salinthone et al. 2007; Arrigo et al. 2007). PLA2 contains several families with diverse enzyme activity. The cytosolic phospholipase A2 (cPLA2) is involved in inflammation and regulated by calcium dependent phosphorylation (Ghosh et al. 2006; Lee and Yang 2013). Increased intracellular calcium concentrations lead to translocation of cPLA2 from the cytosol to the membrane (Glover et al. 1995; Evans et al. 2001). PLA2 is phosphorylated in Serine505 by MAPK and plays a key role in arachidonic acid (AA) during the production of lipid inflammatory mediators (Yedgar et al. 2013). PLC-α plays an important role in the inflammatory responses and pathogenesis of bronchial asthma by upregulating inflammatory cytokine production via bronchial epithelial cells. ApoA-1, which is the major protein component of high density lipoprotein (HDL) in plasma, binds to lipopolysaccharide or endotoxin, leading to anti-endotoxin activity (Ma et al. 2004). ApoA-1 also reduces vascular inflammation and inhibits cholesterol transport from cells. Endogenous ApoA-1 negatively controls the OVA-treated neutrophilic airway inflammation in asthma (Dai et al. 2012). Overall, our results related to those of previous studies indicate that increases in these proteins, lead to airway hyperresponsiveness, and therefore severe asthma. Airway remodeling describes structural changes in airways related asthma, such as thickening of airway walls, airway narrowing, bronchial hyperresponsiveness, and mucous hypersecretion (Fish and Peters 1999). Cytoskeletal proteins are involved in airway remodeling; therefore, elevated expression of those proteins likely contributes to structural changes in airway walls, leading to development of asthma.

Proteome analysis identified 25 upregulated and five downregulated proteins in lung tissues of asthmatic (OVA-treated) mice, some of which were selected for verification as causes of asthma by western blot and qRT-PCR analysis of lung tissue. Several of these proteins have been implicated in inflammatory responses and airway hyperresponsiveness. The elevated CRP and decreased PLA2 in lung tissue are biomarkers for detection of asthma. However, the protein and the mRNA expression of ApoA1 showed the opposite of data of proteomics. Previous studies showed ApoA1 has demonstrate anti-inflammatory activity and preventive effects in asthmatic mouse model (Park et al. 2013). On the other hand, several studies have reported the opposite. ApoA-1 shows pleiotropic anti-inflammatory nature by regulating the innate inflammatory response at the pre-receptor, receptor and post-receptors aspects. The specific post-translational modifications of apoA-1 can transform an anti-inflammatory molecule into a pro-inflammatory one (Vuilleumier et al. 2013). It was considered that the post-translational modified ApoA1 inhibited the anti-inflammation action of normal ApoA1 even though the expression level of ApoA1 was increased in OVA-treated mice. Subsequent studies should address the mechanisms and causes of modification of this protein. It was possible to be increased albumin expression from the increased vessel permeability. In our study, however, some cytokines (Ig G1, Ig E, IL-4, IL-5, and IL-13) in serum and tissues were increased in OVA-treated mice. That meant the immune response was highly triggered by OVA. So, it was considered that the increased albumin was caused by OVA sensitization in our study.

During proteome analysis, we investigated candidates that play crucial roles as biomarkers of asthma by RNA-seq. RNA isolated from whole mouse lung tissue was successfully sequenced, showing transcriptomic profiles of lung tissue that were differentially expressed between controls and asthmatic (OVA-treated) mice. After the lungs are challenged with OVA, many genes show differences in expression and may act as biomarkers that are representative of asthma (Kuperman et al. 2005). We selected 146 genes in lung tissues that were differentially expressed between controls and OVA-treated mice. The level of Clca3 mRNA was significantly elevated in OVA-challenged mice relative to control mice. Clca3 has already been identified as being related to asthma. Many chemokine ligands (ccl8, ccl24, cxcl9, and cxcl10) were shown to be highly elevated in the asthmatic mouse model (Lewis et al. 2008). Expression levels of Chil4 mRNA, which encodes chitinase or chitinase-like protein, were also increased (Shuhui et al. 2009). MMP12 is involved in the breakdown of extracellular matrix, which plays a role in local cellular inflammation in the lung (Lanone et al. 2002). Muc5ac has been linked to mucin production and asthma, and shown to be upregulated in OVA challenge mice (Wang et al. 2012). Retnla, Saa3, Scin, Arg2, and Fcgbp were increased in OVA-challenged mice relative to control mice, may be useful as biomarkers of asthma. Paraoxonase 1 (Pon1) is esterase enzymes displaying antioxidant characteristics. Pon1 is a gene that has characteristics of antioxidant activity. Oxidative stress is involved in the pathogenesis of asthma (Sarioglu et al. 2015). The secretoglobin family 3A member 2 (SCGB3A2) gene is located on chromosome 5 and SCGB3A2 is a small molecular weight secreted protein in airway epithelial cells. This protein has anti-inflammatory activity (Kim et al. 2017). Genetic ablation of neuronal PAS domain protein 2 (Npas2) exaggerates inflammatory responses to lipopolysaccharide and bacterial infection (Gibbs et al. 2014). Flavin containing monooxygenase 3 (FMO3) gene was down regulated in citrobacter rodentrium (Cit) or LPS-induced inflammation model [d]. Elastin gene was reduced in Epithelial-derived inflammation (Zhang et al. 2009). Kruppel-like factor 15 (Klf15) is reduced following mechanical injury or exposure to proproliferative/proinflammatory stimuli (Benjamin et al. 2016). In this study, several genes including Gdpd2, Spon2, Pon1, Scgb3a2, Ighv2-3, Sult1d1, Cyp2f2, Npas2, Arntl, and Igkv4-68 were down-regulated in OVA-challenged mice compared with control mice, may be useful as biomarkers of asthma.

We analyzed genes and proteins differentially expressed in normal and OVA-induced asthmatic mouse lung tissues by 2-D electrophoresis and RNA-seq methods. Most of the identified genes and proteins were involved in inflammation, cytoskeleton, airway remodeling, and metabolism. Recent studies have shown that diverse mechanisms are involved in asthma, including inflammatory activation of asthmatic genes and differential expression of various proteins and cytokines. We identified several genes and proteins involved with asthma in a mouse model, which will facilitate development of novel and effective therapeutic agents for the treatment of allergic asthma.

## Electronic supplementary material

Below is the link to the electronic supplementary material.

Supplementary material 1 (DOCX 64 kb)
