# Supplementary material for: Proteomic and transcriptomic analysis of lung tissue in OVA-challenged mice
Source: Arch Pharm Res. 2017 Oct 30;41(1):87–100. doi: 10.1007/s12272-017-0972-4 (PMC5770490; doi:10.1007/s12272-017-0972-4)
Supplement: Supplementary file 1 — Supplementary material 1 (DOCX 64 kb) [file 12272_2017_972_MOESM1_ESM.docx]

**Online data supplement**

**Proteomic and transcriptomic analysis of lung tissue in OVA-challenged mice**

Yongjin Lee^1, ¶^, Yun-Ho Hwang^1, ¶^, Kwang-Jin Kim^1^, Ae-Kyung Park^1^, Man-Jeong Paik^1^, Seong Hwan Kim^2^, Su Ui Lee^3^, Sung-Tae Yee^1,^ *, and Young-Jin Son^1,^ *

^1^ Department of Pharmacy, Sunchon National University, 255 Jungangno, Suncheon, Jeonnam 57922, Korea

^2^ Laboratory of Translational Therapeutics, Pharmacology Research Center, Division of Drug Discovery Research, Korea Research Institute of Chemical Technology, Daejeon 34114, Korea

^3^Natural Medicine Research Center, Korea Research Institute of Bioscience and Biotechnology, Chungcheongbuk, Cheongju 56212, Korea

*To whom correspondence should be addressed:

Y.-J. S, Phone: +82-61-750-3755, Fax: +82-61-750-3708, E-mail: [sony@sunchon.ac.kr](mailto:sony@sunchon.ac.kr)

S.-T. Y, Phone: +82-61-750-3752, Fax: +82-61-750-3708, E-mail: [sungtae@sunchon.ac.kr](mailto:sungtae@sunchon.ac.kr)

**Table S1. Information of RNA-seq experiments**

| **Tools** | **Version** | **Option** | **Reference**  **(Web-site)** | **Description** |
| --- | --- | --- | --- | --- |
| In house | 2.4.0b | N-ratio of each read > 0.1 and ratio of less than quality 20 (each read) > 0.4 |  | for the quality trimming of sequence reads |
|  |  |  |  |  |
| Star | 2.2.1 |  | http://bioinformatics.oxfordjournals.org/content/early/2012/10/25/bioinformatics.bts635 | ultrafast universal RNA-seq aligner |
| Cufflinks | 0.6.1p1 |  | https://www.ncbi.nlm.nih.gov/pmc/articles/PMC3334321/ | takes the aligned reads from two or more conditions and reports genes and transcripts that are differentially expressed using a rigorous statistical analysis |
| Htseq-count (python lib) | 1.4.0 |  | http://www-huber.embl.de/users/anders/HTSeq/doc/count.html | Counting reads in features |
| TCC |  |  | https://bmcbioinformatics.biomedcentral.com/articles/10.1186/1471-2105-14-219 | for comparing tag count data with robust normalization strategies |
| GO |  |  | http://geneontology.org/ | The GO defines concepts/classes used to describe gene fuction, and relationships between these concepts |

**Table S2. List of differentially expressed genes in OVA-challenged and control mice (q-value <0.05).**

**1) Up-regulated genes**

| **Gene ID** | **Name** | **Control** | **OVA** | **log_2_(^con^/_OVA_)** | **p-value** | **q-value** |
| --- | --- | --- | --- | --- | --- | --- |
| 251823803 | Chil4 | 7.88 | 14446.8 | 10.8 | 0 | 0 |
| 31981459 | Rnase2a | 0 | 530.1 | 8.88 | 0 | 0 |
| 451327622 | Fbp1 | 1.12 | 295.2 | 8.04 | 0 | 0 |
| 251823796 | Capn9 | 0 | 260.1 | 7.85 | 0 | 0 |
| 255918210 | Clca3 | 451.12 | 75920.4 | 7.39 | 0 | 0 |
| 24475796 | Cd209e | 1.12 | 119.7 | 6.73 | 0 | 0 |
| 71896630 | Mrgprg | 1.12 | 111.6 | 6.63 | 0 | 0 |
| 823683768 | Sprr2a3 | 0 | 105.3 | 6.55 | 0 | 0 |
| 103472021 | Retnla | 1333.12 | 78933.6 | 5.89 | 0 | 0 |
| 114431223 | Muc5ac | 106.88 | 5313.6 | 5.64 | 0 | 0 |
| 142351670 | Ccl24 | 16.88 | 829.8 | 5.62 | 0 | 0 |
| 9964289 | Fgf23 | 0 | 45.9 | 5.35 | 0 | 0.05 |
| 576583721 | Gm9522 | 0 | 45.9 | 5.35 | 0 | 0.05 |
| 115392137 | Mmp12 | 70.88 | 2652.3 | 5.23 | 0 | 0 |
| 146134337 | Slc26a4 | 178.88 | 5677.2 | 4.99 | 0 | 0 |
| 71059716 | Cxcl9 | 7.88 | 229.5 | 4.87 | 0 | 0 |
| 1195689586 | Fer1l6 | 94.5 | 2422.8 | 4.68 | 0 | 0 |
| 270341356 | Pla2g4c | 19.12 | 486.9 | 4.67 | 0 | 0 |
| 169790796 | Fcgbp | 229.5 | 5583.6 | 4.6 | 0 | 0 |
| 226246549 | Scin | 28.12 | 680.4 | 4.6 | 0 | 0 |
| 255708468 | Ccl8 | 13.5 | 317.7 | 4.56 | 0 | 0 |
| 371940989 | Cxcl10 | 31.5 | 700.2 | 4.47 | 0 | 0 |
| 21312817 | Ocstamp | 5.62 | 120.6 | 4.42 | 0 | 0 |
| 118130197 | Saa3 | 70.88 | 1328.4 | 4.23 | 0 | 0 |
| 8809767 | Ly6i | 41.62 | 729 | 4.13 | 0 | 0 |
| 118130044 | Agr2 | 34.88 | 597.6 | 4.1 | 0 | 0 |
| 229577225 | Cxcl1 | 7.88 | 127.8 | 4.02 | 0 | 0 |
| 1111696 | AA467197 | 19.12 | 302.4 | 3.98 | 0 | 0 |
| 130502089 | Ackr1 | 7.88 | 114.3 | 3.86 | 0 | 0.01 |
| 157909793 | Ccl11 | 41.62 | 578.7 | 3.8 | 0 | 0 |
| 226958664 | Ccl7 | 11.25 | 142.2 | 3.66 | 0 | 0 |
| 117422438 | Tnfrsf9 | 34.88 | 435.6 | 3.64 | 0 | 0 |
| 1048970731 | Ighg1 | 12.38 | 150.3 | 3.6 | 0 | 0 |
| 12060417 | Arg1 | 47.25 | 567 | 3.58 | 0 | 0 |
| 253795448 | Dbp | 94.5 | 1046.7 | 3.47 | 0 | 0 |
| 324120874 | Itln1 | 9 | 99 | 3.46 | 0 | 0.03 |
| 13991780 | Fxyd4 | 11.25 | 120.6 | 3.42 | 0 | 0.01 |
| 255958165 | Pdcd1lg2 | 18 | 188.1 | 3.39 | 0 | 0 |
| 118130900 | H2-M2 | 20.25 | 209.7 | 3.37 | 0 | 0 |
| 1195738908 | Col6a5 | 42.75 | 440.1 | 3.36 | 0 | 0 |
| 194353956 | Adra2a | 41.62 | 424.8 | 3.35 | 0 | 0 |
| 141803162 | Ccl2 | 21.38 | 217.8 | 3.35 | 0 | 0 |
| 86476055 | Serpina3g | 202.5 | 1957.5 | 3.27 | 0 | 0 |
| 269973938 | Serpina3f | 42.75 | 403.2 | 3.24 | 0 | 0 |
| 27754135 | Chia1 | 964.12 | 8786.7 | 3.19 | 0 | 0 |
| 170650620 | Aldh1a3 | 22.5 | 202.5 | 3.17 | 0 | 0 |
| 146411663 | Tnip3 | 24.75 | 216 | 3.13 | 0 | 0 |
| 673536536 | Timp1 | 104.62 | 884.7 | 3.08 | 0 | 0 |
| 양식의 맨 위  [AU016906](https://www.ncbi.nlm.nih.gov/nuccore/?term=AU016906) | Gm8113 | 66.38 | 562.5 | 3.08 | 0 | 0 |
| 927442694 | Chil3 | 4777.88 | 38766.6 | 3.02 | 0 | 0 |
| 111815141 | Igkv14-126 | 20.25 | 162.9 | 3.01 | 0 | 0.01 |
| 1071929801 | Mx1 | 132.75 | 1031.4 | 2.96 | 0 | 0 |
| 225735578 | Ccl17 | 109.12 | 815.4 | 2.9 | 0 | 0 |
| 149363692 | Apol7c | 27 | 193.5 | 2.84 | 0 | 0.01 |
| 261824022 | Slc5a1 | 127.12 | 844.2 | 2.73 | 0 | 0 |
| 158749627 | Tgtp1 | 109.12 | 707.4 | 2.7 | 0 | 0 |
| 160358822 | Cd5l | 49.5 | 315.9 | 2.67 | 0 | 0 |
| 226693395 | Hmmr | 37.12 | 234.9 | 2.66 | 0 | 0.01 |
| 158631233 | Gatm | 106.88 | 612 | 2.52 | 0 | 0 |
| 145301610 | Ifit1 | 365.62 | 2070 | 2.5 | 0 | 0 |
| 116089295 | Slfn9 | 111.38 | 630 | 2.5 | 0 | 0 |
| 146231982 | Ifit3 | 644.62 | 3626.1 | 2.49 | 0 | 0 |
| 226874850 | Isg15 | 186.75 | 1009.8 | 2.43 | 0 | 0 |
| 118130025 | Ccnb1 | 37.12 | 200.7 | 2.43 | 0 | 0.03 |
| 327412298 | Selp | 131.62 | 693.9 | 2.4 | 0 | 0 |
| 118130193 | Sele | 73.12 | 385.2 | 2.4 | 0 | 0 |
| 71059978 | F10 | 139.5 | 729 | 2.39 | 0 | 0 |
| 13655454 | Ms4a6d | 111.38 | 574.2 | 2.37 | 0 | 0 |
| 2431975 | Ccr5 | 141.75 | 720 | 2.34 | 0 | 0 |
| 928083868 | Aurkb | 46.12 | 233.1 | 2.34 | 0 | 0.02 |
| 34328048 | Lcn2 | 1636.88 | 7838.1 | 2.26 | 0 | 0 |
| 213417961 | Zbp1 | 202.5 | 918 | 2.18 | 0 | 0 |
| 115311550 | Mgl2 | 401.62 | 1782 | 2.15 | 0 | 0 |
| 90093357 | Aspm | 94.5 | 414.9 | 2.13 | 0 | 0.01 |
| 154240695 | Ccl22 | 153 | 659.7 | 2.11 | 0 | 0 |
| 141803421 | Cdk1 | 85.5 | 366.3 | 2.1 | 0 | 0.02 |
| 161621268 | Uhrf1 | 172.12 | 735.3 | 2.09 | 0 | 0 |
| 118344445 | Iqgap3 | 121.5 | 512.1 | 2.08 | 0 | 0.01 |
| 226371765 | Iigp1 | 645.75 | 2719.8 | 2.07 | 0 | 0 |
| 254553433 | Il4i1 | 167.62 | 705.6 | 2.07 | 0 | 0 |
| 153945748 | Top2a | 515.25 | 2150.1 | 2.06 | 0 | 0 |
| 148747281 | Slc6a20a | 88.88 | 366.3 | 2.04 | 0 | 0.02 |
| 6857768 | Ch25h | 153 | 626.4 | 2.03 | 0 | 0.01 |
| 1195721658 | Kntc1 | 85.5 | 350.1 | 2.03 | 0 | 0.03 |
| 354791221 | 2810417H13Rik | 84.38 | 342.9 | 2.02 | 0 | 0.03 |
| 125858490 | C330027C09Rik | 82.12 | 328.5 | 2 | 0 | 0.04 |
| 118129891 | Dok2 | 149.62 | 594 | 1.99 | 0 | 0.01 |
| 118130978 | Ifi204 | 145.12 | 571.5 | 1.98 | 0 | 0.01 |
| 3688606 | Per3 | 698.62 | 2691.9 | 1.95 | 0 | 0 |
| 1195687828 | Nr1d2 | 950.62 | 3587.4 | 1.92 | 0 | 0 |
| 836182218 | Clec7a | 549 | 2078.1 | 1.92 | 0 | 0 |
| 225690578 | Emr4 | 176.62 | 669.6 | 1.92 | 0 | 0.01 |
| 237512932 | Rsad2 | 240.75 | 896.4 | 1.9 | 0 | 0.01 |
| 85540457 | Ccl9 | 344.25 | 1263.6 | 1.88 | 0 | 0 |
| 161353443 | Ccna2 | 160.88 | 591.3 | 1.88 | 0 | 0.02 |
| 45476576 | Kif11 | 151.88 | 560.7 | 1.88 | 0 | 0.02 |
| 1109303224 | Irf4 | 240.75 | 881.1 | 1.87 | 0 | 0.01 |
| 324021655 | Clec4a3 | 149.62 | 534.6 | 1.84 | 0 | 0.03 |
| 357588523 | Irf7 | 478.12 | 1659.6 | 1.8 | 0 | 0.01 |
| 257900494 | Il33 | 1793.25 | 6131.7 | 1.77 | 0 | 0 |
| 213972628 | Tpx2 | 150.75 | 513.9 | 1.77 | 0 | 0.05 |
| 118130089 | Oas3 | 181.12 | 612 | 1.76 | 0 | 0.04 |
| 146260275 | I830012O16Rik | 209.25 | 704.7 | 1.75 | 0 | 0.03 |
| 169234623 | Mki67 | 771.75 | 2547.9 | 1.72 | 0 | 0.01 |
| 281332107 | Oas1a | 264.38 | 872.1 | 1.72 | 0 | 0.02 |
| 357588481 | Tspan4 | 459 | 1498.5 | 1.71 | 0 | 0.01 |
| 160333416 | Ccr2 | 869.62 | 2776.5 | 1.67 | 0 | 0.01 |
| 1195713053 | Ifi44 | 302.62 | 954 | 1.66 | 0 | 0.03 |
| 239985466 | S100a4 | 339.75 | 1064.7 | 1.65 | 0 | 0.03 |
| 530354638 | Ctsk | 464.62 | 1449.9 | 1.64 | 0 | 0.02 |
| 130503300 | Serpina3n | 1613.25 | 5000.4 | 1.63 | 0 | 0.01 |
| 700274131 | Mcm5 | 343.12 | 1062 | 1.63 | 0 | 0.03 |
| 510025484 | Ctss | 2252.25 | 6883.2 | 1.61 | 0 | 0.01 |
| 162461505 | Ifit2 | 643.5 | 1934.1 | 1.59 | 0 | 0.03 |
| 224809460 | Chil1 | 15435 | 46171.8 | 1.58 | 0 | 0.01 |
| 118130485 | Itgax | 1417.5 | 4160.7 | 1.55 | 0 | 0.02 |
| 134053897 | Oasl2 | 1215 | 3530.7 | 1.54 | 0 | 0.03 |
| 1041817914 | Reg3g | 1073.25 | 3094.2 | 1.53 | 0 | 0.03 |

**2) Down-regulated genes**

| **Gene ID** | **Name** | | | **Control** | **OVA** | | **log_2_(^con^/_OVA_)** | **p-value** | | **q-value** |
| --- | --- | --- | --- | --- | --- | --- | --- | --- | --- | --- |
| 959097185 | | Igkv4-68 | 151.88 | | | 10.8 | -3.81 | | 0 | 0 |
| 226234518 | | Ighv2-3 | 199.12 | | | 15.3 | -3.7 | | 0 | 0 |
| 1195742115 | | Gdpd2 | 640.12 | | | 52.2 | -3.62 | | 0 | 0 |
| 양식의 맨 위  6851267 | | Asgr1 | 101.25 | | | 9 | -3.49 | | 0 | 0.02 |
| 양식의 맨 위  254939622 | | Spon2 | 1593 | | | 232.2 | -2.78 | | 0 | 0 |
| 양식의 맨 위  261823994 | | Pon1 | 1292.62 | | | 190.8 | -2.76 | | 0 | 0 |
| 양식의 맨 위  161621276 | | Npas2 | 445.5 | | | 70.2 | -2.67 | | 0 | 0 |
| 양식의 맨 위  188219648 | | Sult1d1 | 625.5 | | | 111.6 | -2.49 | | 0 | 0 |
| 양식의 맨 위  1195729195 | | Scgb1c1 | 186.75 | | | 35.1 | -2.41 | | 0 | 0.04 |
| 양식의 맨 위  576067742 | | Scgb3a2 | 17451 | | | 3397.5 | -2.36 | | 0 | 0 |
| 양식의 맨 위  340007430 | | Arntl | 817.88 | | | 163.8 | -2.32 | | 0 | 0 |
| 양식의 맨 위  146141092 | | Iyd | 209.25 | | | 42.3 | -2.31 | | 0 | 0.04 |
| 양식의 맨 위  922304292 | | Fmo3 | 1608.75 | | | 391.5 | -2.04 | | 0 | 0 |
| 양식의 맨 위  124001559 | | Cyp2f2 | 66559.5 | | | 16542 | -2.01 | | 0 | 0 |
| 양식의 맨 위  163310768 | | Aldh3a1 | 617.62 | | | 161.1 | -1.94 | | 0 | 0.01 |
| 양식의 맨 위  239937452 | | Hmgcs2 | 861.75 | | | 230.4 | -1.9 | | 0 | 0.01 |
| 양식의 맨 위  118130875 | | Pdk4 | 648 | | | 179.1 | -1.86 | | 0 | 0.02 |
| 양식의 맨 위  167830441 | | Dcdc2a | 631.12 | | | 174.6 | -1.85 | | 0 | 0.02 |
| 양식의 맨 위  921274072 | | Eln | 10012.5 | | | 2944.8 | -1.77 | | 0 | 0 |
| 양식의 맨 위  36244607 | | Cyp2a5 | 1776.38 | | | 522.9 | -1.76 | | 0 | 0.01 |
| 양식의 맨 위  118130317 | | Klf15 | 1296 | | | 394.2 | -1.72 | | 0 | 0.01 |
| 양식의 맨 위  118129966 | | Acot1 | 1035 | | | 315.9 | -1.71 | | 0 | 0.02 |
| 양식의 맨 위  227908862 | | Bpifa1 | 13106.2 | | | 4056.3 | -1.69 | | 0 | 0.01 |
| 양식의 맨 위  195346632 | | Gm15883 | 850.5 | | | 262.8 | -1.69 | | 0 | 0.03 |
| 양식의 맨 위  114326498 | | Myl7 | 1294.88 | | | 414 | -1.65 | | 0 | 0.02 |
| 양식의 맨 위  262205572 | | 8430408G22Rik | 949.5 | | | 308.7 | -1.62 | | 0 | 0.04 |
| 양식의 맨 위  255918224 | | Myh6 | 5234.62 | | | 1742.4 | -1.59 | | 0 | 0.02 |
| 양식의 맨 위  134053927 | | Scgb1a1 | 734278 | | | 273020 | -1.43 | | 0 | 0.04 |

**Table S3. List of Gene Ontology Enrichment (cutoff: p-value < 0.001)**

1. **The list of genes involved in molecular function**

| **No.** | **ACC** | **p-value** | **Category** | **# Transcripts** | **Definition** |
| --- | --- | --- | --- | --- | --- |
| 1 | [GO:0008009](file:///K:\mouse_ovalbumin_2016\RNA_TBD150541_20160418\00_report\report\report\GO\molecular_function\GO_0008009.html#S0001-S0002) | 2.413106e-15 | chemokine activity | 11 | chemokine activity |
| 2 | [GO:0042379](file:///K:\mouse_ovalbumin_2016\RNA_TBD150541_20160418\00_report\report\report\GO\molecular_function\GO_0042379.html#S0001-S0002) | 2.469662e-14 | protein binding | 11 | chemokine receptor binding |
| 3 | [GO:0005125](file:///K:\mouse_ovalbumin_2016\RNA_TBD150541_20160418\00_report\report\report\GO\molecular_function\GO_0005125.html#S0001-S0002) | 4.367968e-10 | binding | 13 | cytokine activity |
| 4 | [GO:0001664](file:///K:\mouse_ovalbumin_2016\RNA_TBD150541_20160418\00_report\report\report\GO\molecular_function\GO_0001664.html#S0001-S0002) | 2.468505e-08 | binding | 12 | G-protein coupled receptor binding |
| 5 | [GO:0005126](file:///K:\mouse_ovalbumin_2016\RNA_TBD150541_20160418\00_report\report\report\GO\molecular_function\GO_0005126.html#S0001-S0002) | 2.901433e-07 | binding | 11 | cytokine receptor binding |
| 6 | [GO:0004568](file:///K:\mouse_ovalbumin_2016\RNA_TBD150541_20160418\00_report\report\report\GO\molecular_function\GO_0004568.html#S0001-S0002) | 4.093942e-07 | hydrolase activity | 4 | chitinase activity |
| 7 | [GO:0001730](file:///K:\mouse_ovalbumin_2016\RNA_TBD150541_20160418\00_report\report\report\GO\molecular_function\GO_0001730.html#S0001-S0002) | 1.496575e-06 | nucleotidyltransferase activity | 3 | 2'-5'-oligoadenylate synthetase activity |
| 8 | [GO:0030246](file:///K:\mouse_ovalbumin_2016\RNA_TBD150541_20160418\00_report\report\report\GO\molecular_function\GO_0030246.html#S0001-S0002) | 1.919007e-06 | binding | 11 | carbohydrate binding |
| 9 | [GO:0008061](file:///K:\mouse_ovalbumin_2016\RNA_TBD150541_20160418\00_report\report\report\GO\molecular_function\GO_0008061.html#S0001-S0002) | 5.978633e-06 | chitin binding | 3 | chitin binding |
| 10 | [GO:0019957](file:///K:\mouse_ovalbumin_2016\RNA_TBD150541_20160418\00_report\report\report\GO\molecular_function\GO_0019957.html#S0001-S0002) | 1.151554e-05 | chemokine binding | 3 | C-C chemokine binding |
| 11 | [GO:0005102](file:///K:\mouse_ovalbumin_2016\RNA_TBD150541_20160418\00_report\report\report\GO\molecular_function\GO_0005102.html#S0001-S0002) | 2.208875e-05 | binding | 22 | receptor binding |
| 12 | [GO:0048020](file:///K:\mouse_ovalbumin_2016\RNA_TBD150541_20160418\00_report\report\report\GO\molecular_function\GO_0048020.html#S0001-S0002) | 9.07297e-05 | chemokine receptor binding | 3 | CCR chemokine receptor binding |
| 13 | [GO:0019956](file:///K:\mouse_ovalbumin_2016\RNA_TBD150541_20160418\00_report\report\report\GO\molecular_function\GO_0019956.html#S0001-S0002) | 0.0001341521 | cytokine binding | 3 | chemokine binding |
| 14 | [GO:0031727](file:///K:\mouse_ovalbumin_2016\RNA_TBD150541_20160418\00_report\report\report\GO\molecular_function\GO_0031727.html#S0001-S0002) | 0.0001538093 | chemokine receptor binding | 2 | CCR2 chemokine receptor binding |
| 15 | [GO:0035173](file:///K:\mouse_ovalbumin_2016\RNA_TBD150541_20160418\00_report\report\report\GO\molecular_function\GO_0035173.html#S0001-S0002) | 0.000200128 | phosphotransferase activity, alcohol group as acceptor | 3 | histone kinase activity |
| 16 | [GO:0004028](file:///K:\mouse_ovalbumin_2016\RNA_TBD150541_20160418\00_report\report\report\GO\molecular_function\GO_0004028.html#S0001-S0002) | 0.0003018361 | oxidoreductase activity, acting on the aldehyde or oxo group of donors, NAD or NADP as acceptor | 2 | 3-chloroallyl aldehyde dehydrogenase activity |
| 17 | [GO:0097367](file:///K:\mouse_ovalbumin_2016\RNA_TBD150541_20160418\00_report\report\report\GO\molecular_function\GO_0097367.html#S0001-S0002) | 0.0003269299 | binding | 30 | carbohydrate derivative binding |
| 18 | [GO:0048248](file:///K:\mouse_ovalbumin_2016\RNA_TBD150541_20160418\00_report\report\report\GO\molecular_function\GO_0048248.html#S0001-S0002) | 0.0003363955 | CXCR chemokine receptor binding | 2 | CXCR3 chemokine receptor binding |
| 19 | [GO:0005488](file:///K:\mouse_ovalbumin_2016\RNA_TBD150541_20160418\00_report\report\report\GO\molecular_function\GO_0005488.html#S0001-S0002) | 0.0004769904 | molecular_function | 116 | binding |
| 20 | [GO:0070566](file:///K:\mouse_ovalbumin_2016\RNA_TBD150541_20160418\00_report\report\report\GO\molecular_function\GO_0070566.html#S0001-S0002) | 0.0005306246 | nucleotidyltransferase activity | 3 | adenylyltransferase activity |
| 21 | [GO:0033691](file:///K:\mouse_ovalbumin_2016\RNA_TBD150541_20160418\00_report\report\report\GO\molecular_function\GO_0033691.html#S0001-S0002) | 0.0006826226 | organic acid binding | 2 | sialic acid binding |
| 22 | [GO:0070492](file:///K:\mouse_ovalbumin_2016\RNA_TBD150541_20160418\00_report\report\report\GO\molecular_function\GO_0070492.html#S0001-S0002) | 0.000688523 | carbohydrate binding | 2 | oligosaccharide binding |
| 23 | [GO:0005539](file:///K:\mouse_ovalbumin_2016\RNA_TBD150541_20160418\00_report\report\report\GO\molecular_function\GO_0005539.html#S0001-S0002) | 0.0007576168 | glycosaminoglycan binding | 6 | glycosaminoglycan binding |
| 24 | [GO:0045236](file:///K:\mouse_ovalbumin_2016\RNA_TBD150541_20160418\00_report\report\report\GO\molecular_function\GO_0045236.html#S0001-S0002) | 0.0009589158 | chemokine receptor binding | 2 | CXCR chemokine receptor binding |

1. **The list of genes involved in biological process**

| **No.** | **ACC** | **p-value** | **Category** | **# Transcripts** | **Definition** |
| --- | --- | --- | --- | --- | --- |
| 1 | [GO:0006952](file:///K:\mouse_ovalbumin_2016\RNA_TBD150541_20160418\00_report\report\report\GO\biological_process\GO_0006952.html#S0001-S0002) | 1.837199e-25 | biological_process | 44 | defense response |
| 2 | [GO:0006950](file:///K:\mouse_ovalbumin_2016\RNA_TBD150541_20160418\00_report\report\report\GO\biological_process\GO_0006950.html#S0001-S0002) | 3.809135e-20 | biological_process | 62 | response to stress |
| 3 | [GO:0006955](file:///K:\mouse_ovalbumin_2016\RNA_TBD150541_20160418\00_report\report\report\GO\biological_process\GO_0006955.html#S0001-S0002) | 1.760392e-19 | biological_process | 36 | immune response |
| 4 | [GO:0009611](file:///K:\mouse_ovalbumin_2016\RNA_TBD150541_20160418\00_report\report\report\GO\biological_process\GO_0009611.html#S0001-S0002) | 3.230118e-17 | response to wounding | 33 | response to wounding |
| 5 | [GO:0006954](file:///K:\mouse_ovalbumin_2016\RNA_TBD150541_20160418\00_report\report\report\GO\biological_process\GO_0006954.html#S0001-S0002) | 1.437125e-16 | response to wounding | 26 | inflammatory response |
| 6 | [GO:0002376](file:///K:\mouse_ovalbumin_2016\RNA_TBD150541_20160418\00_report\report\report\GO\biological_process\GO_0002376.html#S0001-S0002) | 4.108041e-16 | immune system process | 45 | immune system process |
| 7 | [GO:0051707](file:///K:\mouse_ovalbumin_2016\RNA_TBD150541_20160418\00_report\report\report\GO\biological_process\GO_0051707.html#S0001-S0002) | 7.985046e-16 | multi-organism process | 28 | response to other organism |
| 8 | [GO:0009607](file:///K:\mouse_ovalbumin_2016\RNA_TBD150541_20160418\00_report\report\report\GO\biological_process\GO_0009607.html#S0001-S0002) | 3.343946e-15 | response to biotic stimulus | 28 | response to biotic stimulus |
| 9 | [GO:0070887](file:///K:\mouse_ovalbumin_2016\RNA_TBD150541_20160418\00_report\report\report\GO\biological_process\GO_0070887.html#S0001-S0002) | 1.136424e-14 | cellular response to stimulus | 43 | cellular response to chemical stimulus |
| 10 | [GO:0034097](file:///K:\mouse_ovalbumin_2016\RNA_TBD150541_20160418\00_report\report\report\GO\biological_process\GO_0034097.html#S0001-S0002) | 1.152404e-14 | response to chemical | 25 | response to cytokine |
| 11 | [GO:0051704](file:///K:\mouse_ovalbumin_2016\RNA_TBD150541_20160418\00_report\report\report\GO\biological_process\GO_0051704.html#S0001-S0002) | 3.220104e-13 | biological_process | 31 | multi-organism process |
| 12 | [GO:0010033](file:///K:\mouse_ovalbumin_2016\RNA_TBD150541_20160418\00_report\report\report\GO\biological_process\GO_0010033.html#S0001-S0002) | 8.78693e-13 | response to chemical | 45 | response to organic substance |
| 13 | [GO:0009615](file:///K:\mouse_ovalbumin_2016\RNA_TBD150541_20160418\00_report\report\report\GO\biological_process\GO_0009615.html#S0001-S0002) | 2.008799e-12 | multi-organism process | 16 | response to virus |
| 14 | [GO:0045087](file:///K:\mouse_ovalbumin_2016\RNA_TBD150541_20160418\00_report\report\report\GO\biological_process\GO_0045087.html#S0001-S0002) | 9.435918e-12 | innate immune response | 19 | innate immune response |
| 15 | [GO:0050896](file:///K:\mouse_ovalbumin_2016\RNA_TBD150541_20160418\00_report\report\report\GO\biological_process\GO_0050896.html#S0001-S0002) | 1.616296e-10 | biological_process | 90 | response to stimulus |
| 16 | [GO:0009605](file:///K:\mouse_ovalbumin_2016\RNA_TBD150541_20160418\00_report\report\report\GO\biological_process\GO_0009605.html#S0001-S0002) | 1.738318e-10 | response to external stimulus | 31 | response to external stimulus |
| 17 | [GO:0042221](file:///K:\mouse_ovalbumin_2016\RNA_TBD150541_20160418\00_report\report\report\GO\biological_process\GO_0042221.html#S0001-S0002) | 2.48835e-10 | response to chemical | 56 | response to chemical |
| 18 | [GO:0071345](file:///K:\mouse_ovalbumin_2016\RNA_TBD150541_20160418\00_report\report\report\GO\biological_process\GO_0071345.html#S0001-S0002) | 2.550661e-10 | cellular response to organic substance | 18 | cellular response to cytokine stimulus |
| 19 | [GO:0098542](file:///K:\mouse_ovalbumin_2016\RNA_TBD150541_20160418\00_report\report\report\GO\biological_process\GO_0098542.html#S0001-S0002) | 3.370913e-10 | response to other organism | 16 | defense response to other organism |
| 20 | [GO:0051607](file:///K:\mouse_ovalbumin_2016\RNA_TBD150541_20160418\00_report\report\report\GO\biological_process\GO_0051607.html#S0001-S0002) | 7.568312e-10 | response to other organism | 12 | defense response to virus |
| 21 | [GO:0002252](file:///K:\mouse_ovalbumin_2016\RNA_TBD150541_20160418\00_report\report\report\GO\biological_process\GO_0002252.html#S0001-S0002) | 8.911533e-10 | immune system process | 19 | immune effector process |
| 22 | [GO:0060326](file:///K:\mouse_ovalbumin_2016\RNA_TBD150541_20160418\00_report\report\report\GO\biological_process\GO_0060326.html#S0001-S0002) | 4.156569e-09 | cell chemotaxis | 12 | cell chemotaxis |
| 23 | [GO:0071310](file:///K:\mouse_ovalbumin_2016\RNA_TBD150541_20160418\00_report\report\report\GO\biological_process\GO_0071310.html#S0001-S0002) | 1.127719e-08 | cellular response to chemical stimulus | 30 | cellular response to organic substance |
| 24 | [GO:0048518](file:///K:\mouse_ovalbumin_2016\RNA_TBD150541_20160418\00_report\report\report\GO\biological_process\GO_0048518.html#S0001-S0002) | 4.836518e-08 | regulation of biological process | 54 | positive regulation of biological process |
| 25 | [GO:0002682](file:///K:\mouse_ovalbumin_2016\RNA_TBD150541_20160418\00_report\report\report\GO\biological_process\GO_0002682.html#S0001-S0002) | 6.179584e-08 | regulation of biological process | 22 | regulation of immune system process |
| 26 | [GO:0002684](file:///K:\mouse_ovalbumin_2016\RNA_TBD150541_20160418\00_report\report\report\GO\biological_process\GO_0002684.html#S0001-S0002) | 1.411586e-07 | regulation of biological process | 17 | positive regulation of immune system process |
| 27 | [GO:0048584](file:///K:\mouse_ovalbumin_2016\RNA_TBD150541_20160418\00_report\report\report\GO\biological_process\GO_0048584.html#S0001-S0002) | 2.89791e-07 | response to stimulus | 27 | positive regulation of response to stimulus |
| 28 | [GO:0035457](file:///K:\mouse_ovalbumin_2016\RNA_TBD150541_20160418\00_report\report\report\GO\biological_process\GO_0035457.html#S0001-S0002) | 3.28186e-07 | cellular response to organic substance | 4 | cellular response to interferon-alpha |
| 29 | [GO:0006030](file:///K:\mouse_ovalbumin_2016\RNA_TBD150541_20160418\00_report\report\report\GO\biological_process\GO_0006030.html#S0001-S0002) | 4.093942e-07 | carbohydrate derivative metabolic process | 4 | chitin metabolic process |
| 30 | [GO:0006032](file:///K:\mouse_ovalbumin_2016\RNA_TBD150541_20160418\00_report\report\report\GO\biological_process\GO_0006032.html#S0001-S0002) | 4.093942e-07 | organonitrogen compound catabolic process | 4 | chitin catabolic process |
| 31 | [GO:0002685](file:///K:\mouse_ovalbumin_2016\RNA_TBD150541_20160418\00_report\report\report\GO\biological_process\GO_0002685.html#S0001-S0002) | 4.25074e-07 | biological regulation | 8 | regulation of leukocyte migration |
| 32 | [GO:0048522](file:///K:\mouse_ovalbumin_2016\RNA_TBD150541_20160418\00_report\report\report\GO\biological_process\GO_0048522.html#S0001-S0002) | 4.299007e-07 | regulation of cellular process | 48 | positive regulation of cellular process |
| 33 | [GO:0031349](file:///K:\mouse_ovalbumin_2016\RNA_TBD150541_20160418\00_report\report\report\GO\biological_process\GO_0031349.html#S0001-S0002) | 5.00907e-07 | biological regulation | 10 | positive regulation of defense response |
| 34 | [GO:0002687](file:///K:\mouse_ovalbumin_2016\RNA_TBD150541_20160418\00_report\report\report\GO\biological_process\GO_0002687.html#S0001-S0002) | 6.353517e-07 | regulation of cell motility | 7 | positive regulation of leukocyte migration |
| 35 | [GO:1901072](file:///K:\mouse_ovalbumin_2016\RNA_TBD150541_20160418\00_report\report\report\GO\biological_process\GO_1901072.html#S0001-S0002) | 6.576693e-07 | carbohydrate derivative catabolic process | 4 | glucosamine-containing compound catabolic process |
| 36 | [GO:0001816](file:///K:\mouse_ovalbumin_2016\RNA_TBD150541_20160418\00_report\report\report\GO\biological_process\GO_0001816.html#S0001-S0002) | 7.261227e-07 | single-organism process | 15 | cytokine production |
| 37 | [GO:0031347](file:///K:\mouse_ovalbumin_2016\RNA_TBD150541_20160418\00_report\report\report\GO\biological_process\GO_0031347.html#S0001-S0002) | 8.422156e-07 | biological regulation | 13 | regulation of defense response |
| 38 | [GO:0001817](file:///K:\mouse_ovalbumin_2016\RNA_TBD150541_20160418\00_report\report\report\GO\biological_process\GO_0001817.html#S0001-S0002) | 8.632693e-07 | regulation of multicellular organismal process | 14 | regulation of cytokine production |
| 39 | [GO:0008284](file:///K:\mouse_ovalbumin_2016\RNA_TBD150541_20160418\00_report\report\report\GO\biological_process\GO_0008284.html#S0001-S0002) | 8.842525e-07 | regulation of cellular process | 18 | positive regulation of cell proliferation |
| 40 | [GO:0051716](file:///K:\mouse_ovalbumin_2016\RNA_TBD150541_20160418\00_report\report\report\GO\biological_process\GO_0051716.html#S0001-S0002) | 1.24426e-06 | response to stimulus | 70 | cellular response to stimulus |
| 41 | [GO:0035455](file:///K:\mouse_ovalbumin_2016\RNA_TBD150541_20160418\00_report\report\report\GO\biological_process\GO_0035455.html#S0001-S0002) | 2.819862e-06 | response to chemical | 4 | response to interferon-alpha |
| 42 | [GO:0071396](file:///K:\mouse_ovalbumin_2016\RNA_TBD150541_20160418\00_report\report\report\GO\biological_process\GO_0071396.html#S0001-S0002) | 3.110013e-06 | cellular response to organic substance | 12 | cellular response to lipid |
| 43 | [GO:0046348](file:///K:\mouse_ovalbumin_2016\RNA_TBD150541_20160418\00_report\report\report\GO\biological_process\GO_0046348.html#S0001-S0002) | 3.64082e-06 | carbohydrate derivative catabolic process | 4 | amino sugar catabolic process |
| 44 | [GO:0009617](file:///K:\mouse_ovalbumin_2016\RNA_TBD150541_20160418\00_report\report\report\GO\biological_process\GO_0009617.html#S0001-S0002) | 3.74502e-06 | multi-organism process | 13 | response to bacterium |
| 45 | [GO:0042127](file:///K:\mouse_ovalbumin_2016\RNA_TBD150541_20160418\00_report\report\report\GO\biological_process\GO_0042127.html#S0001-S0002) | 5.556273e-06 | regulation of cellular process | 23 | regulation of cell proliferation |
| 46 | [GO:0016477](file:///K:\mouse_ovalbumin_2016\RNA_TBD150541_20160418\00_report\report\report\GO\biological_process\GO_0016477.html#S0001-S0002) | 5.588282e-06 | localization | 20 | cell migration |
| 47 | [GO:0006935](file:///K:\mouse_ovalbumin_2016\RNA_TBD150541_20160418\00_report\report\report\GO\biological_process\GO_0006935.html#S0001-S0002) | 6.185689e-06 | taxis | 13 | chemotaxis |
| 48 | [GO:0042330](file:///K:\mouse_ovalbumin_2016\RNA_TBD150541_20160418\00_report\report\report\GO\biological_process\GO_0042330.html#S0001-S0002) | 6.358844e-06 | taxis | 13 | taxis |
| 49 | [GO:0070098](file:///K:\mouse_ovalbumin_2016\RNA_TBD150541_20160418\00_report\report\report\GO\biological_process\GO_0070098.html#S0001-S0002) | 8.05993e-06 | cellular response to organic substance | 5 | chemokine-mediated signaling pathway |
| 50 | [GO:0008283](file:///K:\mouse_ovalbumin_2016\RNA_TBD150541_20160418\00_report\report\report\GO\biological_process\GO_0008283.html#S0001-S0002) | 9.180557e-06 | cell proliferation | 26 | cell proliferation |
| 51 | [GO:0050900](file:///K:\mouse_ovalbumin_2016\RNA_TBD150541_20160418\00_report\report\report\GO\biological_process\GO_0050900.html#S0001-S0002) | 9.572006e-06 | localization | 9 | leukocyte migration |
| 52 | [GO:1901700](file:///K:\mouse_ovalbumin_2016\RNA_TBD150541_20160418\00_report\report\report\GO\biological_process\GO_1901700.html#S0001-S0002) | 9.739257e-06 | response to stimulus | 22 | response to oxygen-containing compound |
| 53 | [GO:0051239](file:///K:\mouse_ovalbumin_2016\RNA_TBD150541_20160418\00_report\report\report\GO\biological_process\GO_0051239.html#S0001-S0002) | 1.247665e-05 | regulation of multicellular organismal process | 33 | regulation of multicellular organismal process |
| 54 | [GO:0048245](file:///K:\mouse_ovalbumin_2016\RNA_TBD150541_20160418\00_report\report\report\GO\biological_process\GO_0048245.html#S0001-S0002) | 1.248514e-05 | myeloid leukocyte migration | 3 | eosinophil chemotaxis |
| 55 | [GO:0048247](file:///K:\mouse_ovalbumin_2016\RNA_TBD150541_20160418\00_report\report\report\GO\biological_process\GO_0048247.html#S0001-S0002) | 1.323274e-05 | cellular response to chemical stimulus | 4 | lymphocyte chemotaxis |
| 56 | [GO:0001819](file:///K:\mouse_ovalbumin_2016\RNA_TBD150541_20160418\00_report\report\report\GO\biological_process\GO_0001819.html#S0001-S0002) | 1.611572e-05 | positive regulation of multicellular organismal process | 9 | positive regulation of cytokine production |
| 57 | [GO:0048870](file:///K:\mouse_ovalbumin_2016\RNA_TBD150541_20160418\00_report\report\report\GO\biological_process\GO_0048870.html#S0001-S0002) | 1.744315e-05 | localization | 20 | cell motility |
| 58 | [GO:0051674](file:///K:\mouse_ovalbumin_2016\RNA_TBD150541_20160418\00_report\report\report\GO\biological_process\GO_0051674.html#S0001-S0002) | 1.744315e-05 | localization | 20 | localization of cell |
| 59 | [GO:0080134](file:///K:\mouse_ovalbumin_2016\RNA_TBD150541_20160418\00_report\report\report\GO\biological_process\GO_0080134.html#S0001-S0002) | 1.972647e-05 | biological regulation | 17 | regulation of response to stress |
| 60 | [GO:0006026](file:///K:\mouse_ovalbumin_2016\RNA_TBD150541_20160418\00_report\report\report\GO\biological_process\GO_0006026.html#S0001-S0002) | 2.582853e-05 | organonitrogen compound catabolic process | 4 | aminoglycan catabolic process |
| 61 | [GO:0033993](file:///K:\mouse_ovalbumin_2016\RNA_TBD150541_20160418\00_report\report\report\GO\biological_process\GO_0033993.html#S0001-S0002) | 2.74122e-05 | response to chemical | 16 | response to lipid |
| 62 | [GO:0044699](file:///K:\mouse_ovalbumin_2016\RNA_TBD150541_20160418\00_report\report\report\GO\biological_process\GO_0044699.html#S0001-S0002) | 2.826896e-05 | biological_process | 115 | single-organism process |
| 63 | [GO:1901071](file:///K:\mouse_ovalbumin_2016\RNA_TBD150541_20160418\00_report\report\report\GO\biological_process\GO_1901071.html#S0001-S0002) | 2.979253e-05 | glucosamine-containing compound metabolic process | 4 | glucosamine-containing compound metabolic process |
| 64 | [GO:0070741](file:///K:\mouse_ovalbumin_2016\RNA_TBD150541_20160418\00_report\report\report\GO\biological_process\GO_0070741.html#S0001-S0002) | 3.233014e-05 | response to stimulus | 4 | response to interleukin-6 |
| 65 | [GO:0009725](file:///K:\mouse_ovalbumin_2016\RNA_TBD150541_20160418\00_report\report\report\GO\biological_process\GO_0009725.html#S0001-S0002) | 3.266223e-05 | response to chemical | 16 | response to hormone |
| 66 | [GO:0002221](file:///K:\mouse_ovalbumin_2016\RNA_TBD150541_20160418\00_report\report\report\GO\biological_process\GO_0002221.html#S0001-S0002) | 3.314984e-05 | biological regulation | 6 | pattern recognition receptor signaling pathway |
| 67 | [GO:0045123](file:///K:\mouse_ovalbumin_2016\RNA_TBD150541_20160418\00_report\report\report\GO\biological_process\GO_0045123.html#S0001-S0002) | 3.848002e-05 | localization | 4 | cellular extravasation |
| 68 | [GO:0002758](file:///K:\mouse_ovalbumin_2016\RNA_TBD150541_20160418\00_report\report\report\GO\biological_process\GO_0002758.html#S0001-S0002) | 4.008975e-05 | biological regulation | 6 | innate immune response-activating signal transduction |
| 69 | [GO:0002434](file:///K:\mouse_ovalbumin_2016\RNA_TBD150541_20160418\00_report\report\report\GO\biological_process\GO_0002434.html#S0001-S0002) | 4.032225e-05 | immune complex clearance | 2 | immune complex clearance |
| 70 | [GO:0002436](file:///K:\mouse_ovalbumin_2016\RNA_TBD150541_20160418\00_report\report\report\GO\biological_process\GO_0002436.html#S0001-S0002) | 4.032225e-05 | immune complex clearance by monocytes and macrophages | 2 | immune complex clearance by monocytes and macrophages |
| 71 | [GO:0090264](file:///K:\mouse_ovalbumin_2016\RNA_TBD150541_20160418\00_report\report\report\GO\biological_process\GO_0090264.html#S0001-S0002) | 4.032225e-05 | biological regulation | 2 | regulation of immune complex clearance by monocytes and macrophages |
| 72 | [GO:0090265](file:///K:\mouse_ovalbumin_2016\RNA_TBD150541_20160418\00_report\report\report\GO\biological_process\GO_0090265.html#S0001-S0002) | 4.032225e-05 | regulation of immune complex clearance by monocytes and macrophages | 2 | positive regulation of immune complex clearance by monocytes and macrophages |
| 73 | [GO:0072677](file:///K:\mouse_ovalbumin_2016\RNA_TBD150541_20160418\00_report\report\report\GO\biological_process\GO_0072677.html#S0001-S0002) | 4.194891e-05 | myeloid leukocyte migration | 3 | eosinophil migration |
| 74 | [GO:2000401](file:///K:\mouse_ovalbumin_2016\RNA_TBD150541_20160418\00_report\report\report\GO\biological_process\GO_2000401.html#S0001-S0002) | 4.348527e-05 | regulation of cell motility | 4 | regulation of lymphocyte migration |
| 75 | [GO:0032656](file:///K:\mouse_ovalbumin_2016\RNA_TBD150541_20160418\00_report\report\report\GO\biological_process\GO_0032656.html#S0001-S0002) | 4.459227e-05 | regulation of multicellular organismal process | 3 | regulation of interleukin-13 production |
| 76 | [GO:0032616](file:///K:\mouse_ovalbumin_2016\RNA_TBD150541_20160418\00_report\report\report\GO\biological_process\GO_0032616.html#S0001-S0002) | 5.364089e-05 | single-organism process | 3 | interleukin-13 production |
| 77 | [GO:0009636](file:///K:\mouse_ovalbumin_2016\RNA_TBD150541_20160418\00_report\report\report\GO\biological_process\GO_0009636.html#S0001-S0002) | 5.372403e-05 | response to chemical | 7 | response to toxic substance |
| 78 | [GO:0050679](file:///K:\mouse_ovalbumin_2016\RNA_TBD150541_20160418\00_report\report\report\GO\biological_process\GO_0050679.html#S0001-S0002) | 5.720631e-05 | regulation of cellular process | 7 | positive regulation of epithelial cell proliferation |
| 79 | [GO:0032496](file:///K:\mouse_ovalbumin_2016\RNA_TBD150541_20160418\00_report\report\report\GO\biological_process\GO_0032496.html#S0001-S0002) | 5.960674e-05 | response to other organism | 9 | response to lipopolysaccharide |
| 80 | [GO:0002218](file:///K:\mouse_ovalbumin_2016\RNA_TBD150541_20160418\00_report\report\report\GO\biological_process\GO_0002218.html#S0001-S0002) | 6.185761e-05 | biological regulation | 6 | activation of innate immune response |
| 81 | [GO:1901652](file:///K:\mouse_ovalbumin_2016\RNA_TBD150541_20160418\00_report\report\report\GO\biological_process\GO_1901652.html#S0001-S0002) | 6.449306e-05 | response to nitrogen compound | 10 | response to peptide |
| 82 | [GO:0009991](file:///K:\mouse_ovalbumin_2016\RNA_TBD150541_20160418\00_report\report\report\GO\biological_process\GO_0009991.html#S0001-S0002) | 6.606571e-05 | response to extracellular stimulus | 11 | response to extracellular stimulus |
| 83 | [GO:0071216](file:///K:\mouse_ovalbumin_2016\RNA_TBD150541_20160418\00_report\report\report\GO\biological_process\GO_0071216.html#S0001-S0002) | 7.082388e-05 | cellular response to stimulus | 7 | cellular response to biotic stimulus |
| 84 | [GO:0040011](file:///K:\mouse_ovalbumin_2016\RNA_TBD150541_20160418\00_report\report\report\GO\biological_process\GO_0040011.html#S0001-S0002) | 7.322545e-05 | biological_process | 21 | locomotion |
| 85 | [GO:0090224](file:///K:\mouse_ovalbumin_2016\RNA_TBD150541_20160418\00_report\report\report\GO\biological_process\GO_0090224.html#S0001-S0002) | 7.616396e-05 | cellular component organization or biogenesis | 3 | regulation of spindle organization |
| 86 | [GO:0032103](file:///K:\mouse_ovalbumin_2016\RNA_TBD150541_20160418\00_report\report\report\GO\biological_process\GO_0032103.html#S0001-S0002) | 8.877991e-05 | response to stimulus | 7 | positive regulation of response to external stimulus |
| 87 | [GO:0002237](file:///K:\mouse_ovalbumin_2016\RNA_TBD150541_20160418\00_report\report\report\GO\biological_process\GO_0002237.html#S0001-S0002) | 9.382129e-05 | multi-organism process | 9 | response to molecule of bacterial origin |
| 88 | [GO:0006928](file:///K:\mouse_ovalbumin_2016\RNA_TBD150541_20160418\00_report\report\report\GO\biological_process\GO_0006928.html#S0001-S0002) | 9.422515e-05 | single-organism process | 22 | cellular component movement |
| 89 | [GO:1901623](file:///K:\mouse_ovalbumin_2016\RNA_TBD150541_20160418\00_report\report\report\GO\biological_process\GO_1901623.html#S0001-S0002) | 9.771642e-05 | regulation of cell motility | 3 | regulation of lymphocyte chemotaxis |
| 90 | [GO:0002224](file:///K:\mouse_ovalbumin_2016\RNA_TBD150541_20160418\00_report\report\report\GO\biological_process\GO_0002224.html#S0001-S0002) | 9.900554e-05 | biological regulation | 5 | toll-like receptor signaling pathway |
| 91 | [GO:0045088](file:///K:\mouse_ovalbumin_2016\RNA_TBD150541_20160418\00_report\report\report\GO\biological_process\GO_0045088.html#S0001-S0002) | 0.0001284128 | biological regulation | 7 | regulation of innate immune response |
| 92 | [GO:0030335](file:///K:\mouse_ovalbumin_2016\RNA_TBD150541_20160418\00_report\report\report\GO\biological_process\GO_0030335.html#S0001-S0002) | 0.0001360273 | regulation of cell motility | 9 | positive regulation of cell migration |
| 93 | [GO:0007584](file:///K:\mouse_ovalbumin_2016\RNA_TBD150541_20160418\00_report\report\report\GO\biological_process\GO_0007584.html#S0001-S0002) | 0.0001429786 | response to chemical | 7 | response to nutrient |
| 94 | [GO:0007052](file:///K:\mouse_ovalbumin_2016\RNA_TBD150541_20160418\00_report\report\report\GO\biological_process\GO_0007052.html#S0001-S0002) | 0.000146581 | protein complex subunit organization | 4 | mitotic spindle organization |
| 95 | [GO:2000147](file:///K:\mouse_ovalbumin_2016\RNA_TBD150541_20160418\00_report\report\report\GO\biological_process\GO_2000147.html#S0001-S0002) | 0.0001571383 | regulation of cell motility | 9 | positive regulation of cell motility |
| 96 | [GO:0031667](file:///K:\mouse_ovalbumin_2016\RNA_TBD150541_20160418\00_report\report\report\GO\biological_process\GO_0031667.html#S0001-S0002) | 0.00016531 | response to nutrient levels | 10 | response to nutrient levels |
| 97 | [GO:1901701](file:///K:\mouse_ovalbumin_2016\RNA_TBD150541_20160418\00_report\report\report\GO\biological_process\GO_1901701.html#S0001-S0002) | 0.0001662722 | response to oxygen-containing compound | 14 | cellular response to oxygen-containing compound |
| 98 | [GO:0045069](file:///K:\mouse_ovalbumin_2016\RNA_TBD150541_20160418\00_report\report\report\GO\biological_process\GO_0045069.html#S0001-S0002) | 0.0001731834 | multi-organism process | 4 | regulation of viral genome replication |
| 99 | [GO:0035747](file:///K:\mouse_ovalbumin_2016\RNA_TBD150541_20160418\00_report\report\report\GO\biological_process\GO_0035747.html#S0001-S0002) | 0.000177113 | cellular response to chemical stimulus | 2 | natural killer cell chemotaxis |
| 100 | [GO:2000501](file:///K:\mouse_ovalbumin_2016\RNA_TBD150541_20160418\00_report\report\report\GO\biological_process\GO_2000501.html#S0001-S0002) | 0.000177113 | regulation of lymphocyte migration | 2 | regulation of natural killer cell chemotaxis |
| 101 | [GO:0051272](file:///K:\mouse_ovalbumin_2016\RNA_TBD150541_20160418\00_report\report\report\GO\biological_process\GO_0051272.html#S0001-S0002) | 0.0001893701 | positive regulation of cellular component movement | 9 | positive regulation of cellular component movement |
| 102 | [GO:0071222](file:///K:\mouse_ovalbumin_2016\RNA_TBD150541_20160418\00_report\report\report\GO\biological_process\GO_0071222.html#S0001-S0002) | 0.000191891 | response to oxygen-containing compound | 6 | cellular response to lipopolysaccharide |
| 103 | [GO:0043434](file:///K:\mouse_ovalbumin_2016\RNA_TBD150541_20160418\00_report\report\report\GO\biological_process\GO_0043434.html#S0001-S0002) | 0.0001977249 | response to nitrogen compound | 9 | response to peptide hormone |
| 104 | [GO:0014070](file:///K:\mouse_ovalbumin_2016\RNA_TBD150541_20160418\00_report\report\report\GO\biological_process\GO_0014070.html#S0001-S0002) | 0.0002054551 | response to chemical | 15 | response to organic cyclic compound |
| 105 | [GO:0070555](file:///K:\mouse_ovalbumin_2016\RNA_TBD150541_20160418\00_report\report\report\GO\biological_process\GO_0070555.html#S0001-S0002) | 0.0002143487 | response to stimulus | 5 | response to interleukin-1 |
| 106 | [GO:0030595](file:///K:\mouse_ovalbumin_2016\RNA_TBD150541_20160418\00_report\report\report\GO\biological_process\GO_0030595.html#S0001-S0002) | 0.0002217713 | cell chemotaxis | 6 | leukocyte chemotaxis |
| 107 | [GO:0072676](file:///K:\mouse_ovalbumin_2016\RNA_TBD150541_20160418\00_report\report\report\GO\biological_process\GO_0072676.html#S0001-S0002) | 0.0002223442 | localization of cell | 4 | lymphocyte migration |
| 108 | [GO:0006040](file:///K:\mouse_ovalbumin_2016\RNA_TBD150541_20160418\00_report\report\report\GO\biological_process\GO_0006040.html#S0001-S0002) | 0.0002343881 | organic substance metabolic process | 4 | amino sugar metabolic process |
| 109 | [GO:0070207](file:///K:\mouse_ovalbumin_2016\RNA_TBD150541_20160418\00_report\report\report\GO\biological_process\GO_0070207.html#S0001-S0002) | 0.0002425104 | protein complex subunit organization | 3 | protein homotrimerization |
| 110 | [GO:0032879](file:///K:\mouse_ovalbumin_2016\RNA_TBD150541_20160418\00_report\report\report\GO\biological_process\GO_0032879.html#S0001-S0002) | 0.0002453855 | localization | 25 | regulation of localization |
| 111 | [GO:0071385](file:///K:\mouse_ovalbumin_2016\RNA_TBD150541_20160418\00_report\report\report\GO\biological_process\GO_0071385.html#S0001-S0002) | 0.000250794 | cellular response to organic cyclic compound | 4 | cellular response to glucocorticoid stimulus |
| 112 | [GO:0040017](file:///K:\mouse_ovalbumin_2016\RNA_TBD150541_20160418\00_report\report\report\GO\biological_process\GO_0040017.html#S0001-S0002) | 0.0002599086 | regulation of biological process | 9 | positive regulation of locomotion |
| 113 | [GO:0072683](file:///K:\mouse_ovalbumin_2016\RNA_TBD150541_20160418\00_report\report\report\GO\biological_process\GO_0072683.html#S0001-S0002) | 0.0002727667 | T cell migration | 2 | T cell extravasation |
| 114 | [GO:0048583](file:///K:\mouse_ovalbumin_2016\RNA_TBD150541_20160418\00_report\report\report\GO\biological_process\GO_0048583.html#S0001-S0002) | 0.0002736788 | response to stimulus | 34 | regulation of response to stimulus |
| 115 | [GO:0071384](file:///K:\mouse_ovalbumin_2016\RNA_TBD150541_20160418\00_report\report\report\GO\biological_process\GO_0071384.html#S0001-S0002) | 0.0002749155 | cellular response to organic cyclic compound | 4 | cellular response to corticosteroid stimulus |
| 116 | [GO:0071219](file:///K:\mouse_ovalbumin_2016\RNA_TBD150541_20160418\00_report\report\report\GO\biological_process\GO_0071219.html#S0001-S0002) | 0.0002773242 | cellular response to biotic stimulus | 6 | cellular response to molecule of bacterial origin |
| 117 | [GO:0050776](file:///K:\mouse_ovalbumin_2016\RNA_TBD150541_20160418\00_report\report\report\GO\biological_process\GO_0050776.html#S0001-S0002) | 0.0002846656 | response to stimulus | 11 | regulation of immune response |
| 118 | [GO:0045089](file:///K:\mouse_ovalbumin_2016\RNA_TBD150541_20160418\00_report\report\report\GO\biological_process\GO_0045089.html#S0001-S0002) | 0.0002871799 | biological regulation | 6 | positive regulation of innate immune response |
| 119 | [GO:0043146](file:///K:\mouse_ovalbumin_2016\RNA_TBD150541_20160418\00_report\report\report\GO\biological_process\GO_0043146.html#S0001-S0002) | 0.00028844 | regulation of protein depolymerization | 2 | spindle stabilization |
| 120 | [GO:0019221](file:///K:\mouse_ovalbumin_2016\RNA_TBD150541_20160418\00_report\report\report\GO\biological_process\GO_0019221.html#S0001-S0002) | 0.000293644 | cellular response to organic substance | 8 | cytokine-mediated signaling pathway |
| 121 | [GO:0042060](file:///K:\mouse_ovalbumin_2016\RNA_TBD150541_20160418\00_report\report\report\GO\biological_process\GO_0042060.html#S0001-S0002) | 0.0003034716 | wound healing | 9 | wound healing |
| 122 | [GO:0031099](file:///K:\mouse_ovalbumin_2016\RNA_TBD150541_20160418\00_report\report\report\GO\biological_process\GO_0031099.html#S0001-S0002) | 0.0003123038 | single-organism developmental process | 6 | regeneration |
| 123 | [GO:0033273](file:///K:\mouse_ovalbumin_2016\RNA_TBD150541_20160418\00_report\report\report\GO\biological_process\GO_0033273.html#S0001-S0002) | 0.0003182598 | response to chemical | 5 | response to vitamin |
| 124 | [GO:0019079](file:///K:\mouse_ovalbumin_2016\RNA_TBD150541_20160418\00_report\report\report\GO\biological_process\GO_0019079.html#S0001-S0002) | 0.00032412 | multi-organism cellular process | 4 | viral genome replication |
| 125 | [GO:0034340](file:///K:\mouse_ovalbumin_2016\RNA_TBD150541_20160418\00_report\report\report\GO\biological_process\GO_0034340.html#S0001-S0002) | 0.0003247086 | innate immune response | 3 | response to type I interferon |
| 126 | [GO:0002690](file:///K:\mouse_ovalbumin_2016\RNA_TBD150541_20160418\00_report\report\report\GO\biological_process\GO_0002690.html#S0001-S0002) | 0.0003449101 | regulation of cell motility | 4 | positive regulation of leukocyte chemotaxis |
| 127 | [GO:2000403](file:///K:\mouse_ovalbumin_2016\RNA_TBD150541_20160418\00_report\report\report\GO\biological_process\GO_2000403.html#S0001-S0002) | 0.0003682865 | regulation of lymphocyte migration | 3 | positive regulation of lymphocyte migration |
| 128 | [GO:0032673](file:///K:\mouse_ovalbumin_2016\RNA_TBD150541_20160418\00_report\report\report\GO\biological_process\GO_0032673.html#S0001-S0002) | 0.0004137259 | regulation of multicellular organismal process | 3 | regulation of interleukin-4 production |
| 129 | [GO:0071383](file:///K:\mouse_ovalbumin_2016\RNA_TBD150541_20160418\00_report\report\report\GO\biological_process\GO_0071383.html#S0001-S0002) | 0.000425469 | cellular response to organic cyclic compound | 6 | cellular response to steroid hormone stimulus |
| 130 | [GO:0044763](file:///K:\mouse_ovalbumin_2016\RNA_TBD150541_20160418\00_report\report\report\GO\biological_process\GO_0044763.html#S0001-S0002) | 0.0004411202 | single-organism process | 104 | single-organism cellular process |
| 131 | [GO:0071354](file:///K:\mouse_ovalbumin_2016\RNA_TBD150541_20160418\00_report\report\report\GO\biological_process\GO_0071354.html#S0001-S0002) | 0.000460464 | cellular response to cytokine stimulus | 3 | cellular response to interleukin-6 |
| 132 | [GO:0035458](file:///K:\mouse_ovalbumin_2016\RNA_TBD150541_20160418\00_report\report\report\GO\biological_process\GO_0035458.html#S0001-S0002) | 0.0004767704 | cellular response to organic substance | 3 | cellular response to interferon-beta |
| 133 | [GO:0002828](file:///K:\mouse_ovalbumin_2016\RNA_TBD150541_20160418\00_report\report\report\GO\biological_process\GO_0002828.html#S0001-S0002) | 0.000478497 | response to stimulus | 3 | regulation of type 2 immune response |
| 134 | [GO:0009719](file:///K:\mouse_ovalbumin_2016\RNA_TBD150541_20160418\00_report\report\report\GO\biological_process\GO_0009719.html#S0001-S0002) | 0.000486378 | response to endogenous stimulus | 19 | response to endogenous stimulus |
| 135 | [GO:0006022](file:///K:\mouse_ovalbumin_2016\RNA_TBD150541_20160418\00_report\report\report\GO\biological_process\GO_0006022.html#S0001-S0002) | 0.0004920156 | carbohydrate derivative metabolic process | 5 | aminoglycan metabolic process |
| 136 | [GO:0051384](file:///K:\mouse_ovalbumin_2016\RNA_TBD150541_20160418\00_report\report\report\GO\biological_process\GO_0051384.html#S0001-S0002) | 0.0004929312 | response to stimulus | 6 | response to glucocorticoid |
| 137 | [GO:0051048](file:///K:\mouse_ovalbumin_2016\RNA_TBD150541_20160418\00_report\report\report\GO\biological_process\GO_0051048.html#S0001-S0002) | 0.0005019578 | establishment of localization | 6 | negative regulation of secretion |
| 138 | [GO:0007093](file:///K:\mouse_ovalbumin_2016\RNA_TBD150541_20160418\00_report\report\report\GO\biological_process\GO_0007093.html#S0001-S0002) | 0.0005471107 | regulation of mitotic cell cycle phase transition | 5 | mitotic cell cycle checkpoint |
| 139 | [GO:0001938](file:///K:\mouse_ovalbumin_2016\RNA_TBD150541_20160418\00_report\report\report\GO\biological_process\GO_0001938.html#S0001-S0002) | 0.000572531 | regulation of cellular process | 4 | positive regulation of endothelial cell proliferation |
| 140 | [GO:0050778](file:///K:\mouse_ovalbumin_2016\RNA_TBD150541_20160418\00_report\report\report\GO\biological_process\GO_0050778.html#S0001-S0002) | 0.0005728525 | response to stimulus | 9 | positive regulation of immune response |
| 141 | [GO:0010193](file:///K:\mouse_ovalbumin_2016\RNA_TBD150541_20160418\00_report\report\report\GO\biological_process\GO_0010193.html#S0001-S0002) | 0.0005794592 | response to stimulus | 2 | response to ozone |
| 142 | [GO:0030334](file:///K:\mouse_ovalbumin_2016\RNA_TBD150541_20160418\00_report\report\report\GO\biological_process\GO_0030334.html#S0001-S0002) | 0.0006132191 | biological regulation | 11 | regulation of cell migration |
| 143 | [GO:0035456](file:///K:\mouse_ovalbumin_2016\RNA_TBD150541_20160418\00_report\report\report\GO\biological_process\GO_0035456.html#S0001-S0002) | 0.0006468578 | response to chemical | 3 | response to interferon-beta |
| 144 | [GO:0050729](file:///K:\mouse_ovalbumin_2016\RNA_TBD150541_20160418\00_report\report\report\GO\biological_process\GO_0050729.html#S0001-S0002) | 0.0006536874 | biological regulation | 4 | positive regulation of inflammatory response |
| 145 | [GO:0032101](file:///K:\mouse_ovalbumin_2016\RNA_TBD150541_20160418\00_report\report\report\GO\biological_process\GO_0032101.html#S0001-S0002) | 0.0006613027 | response to stimulus | 10 | regulation of response to external stimulus |
| 146 | [GO:0002693](file:///K:\mouse_ovalbumin_2016\RNA_TBD150541_20160418\00_report\report\report\GO\biological_process\GO_0002693.html#S0001-S0002) | 0.000714086 | regulation of cell motility | 2 | positive regulation of cellular extravasation |
| 147 | [GO:0031960](file:///K:\mouse_ovalbumin_2016\RNA_TBD150541_20160418\00_report\report\report\GO\biological_process\GO_0031960.html#S0001-S0002) | 0.0007298534 | response to steroid hormone | 6 | response to corticosteroid |
| 148 | [GO:0097529](file:///K:\mouse_ovalbumin_2016\RNA_TBD150541_20160418\00_report\report\report\GO\biological_process\GO_0097529.html#S0001-S0002) | 0.0007534651 | localization of cell | 5 | myeloid leukocyte migration |
| 149 | [GO:0045071](file:///K:\mouse_ovalbumin_2016\RNA_TBD150541_20160418\00_report\report\report\GO\biological_process\GO_0045071.html#S0001-S0002) | 0.0007699535 | multi-organism process | 3 | negative regulation of viral genome replication |
| 150 | [GO:0002756](file:///K:\mouse_ovalbumin_2016\RNA_TBD150541_20160418\00_report\report\report\GO\biological_process\GO_0002756.html#S0001-S0002) | 0.0007852693 | biological regulation | 2 | MyD88-independent toll-like receptor signaling pathway |
| 151 | [GO:0042092](file:///K:\mouse_ovalbumin_2016\RNA_TBD150541_20160418\00_report\report\report\GO\biological_process\GO_0042092.html#S0001-S0002) | 0.0008028556 | type 2 immune response | 3 | type 2 immune response |
| 152 | [GO:0002688](file:///K:\mouse_ovalbumin_2016\RNA_TBD150541_20160418\00_report\report\report\GO\biological_process\GO_0002688.html#S0001-S0002) | 0.0008384256 | cellular response to chemical stimulus | 4 | regulation of leukocyte chemotaxis |
| 153 | [GO:2000145](file:///K:\mouse_ovalbumin_2016\RNA_TBD150541_20160418\00_report\report\report\GO\biological_process\GO_2000145.html#S0001-S0002) | 0.0008397258 | biological regulation | 11 | regulation of cell motility |
| 154 | [GO:0032963](file:///K:\mouse_ovalbumin_2016\RNA_TBD150541_20160418\00_report\report\report\GO\biological_process\GO_0032963.html#S0001-S0002) | 0.0008474688 | single-organism metabolic process | 4 | collagen metabolic process |
| 155 | [GO:0016572](file:///K:\mouse_ovalbumin_2016\RNA_TBD150541_20160418\00_report\report\report\GO\biological_process\GO_0016572.html#S0001-S0002) | 0.000864425 | organic substance metabolic process | 3 | histone phosphorylation |
| 156 | [GO:0031100](file:///K:\mouse_ovalbumin_2016\RNA_TBD150541_20160418\00_report\report\report\GO\biological_process\GO_0031100.html#S0001-S0002) | 0.0008737764 | system development | 4 | organ regeneration |
| 157 | [GO:0032633](file:///K:\mouse_ovalbumin_2016\RNA_TBD150541_20160418\00_report\report\report\GO\biological_process\GO_0032633.html#S0001-S0002) | 0.000889963 | single-organism process | 3 | interleukin-4 production |
| 158 | [GO:0044764](file:///K:\mouse_ovalbumin_2016\RNA_TBD150541_20160418\00_report\report\report\GO\biological_process\GO_0044764.html#S0001-S0002) | 0.0009203485 | multi-organism cellular process | 6 | multi-organism cellular process |
| 159 | [GO:0044259](file:///K:\mouse_ovalbumin_2016\RNA_TBD150541_20160418\00_report\report\report\GO\biological_process\GO_0044259.html#S0001-S0002) | 0.000963992 | single-organism metabolic process | 4 | multicellular organismal macromolecule metabolic process |

1. **The list of genes involved in cellular component**

| **No.** | **ACC** | **p-value** | **Category** | **# Transcripts** | **Definition** |
| --- | --- | --- | --- | --- | --- |
| 1 | [GO:0005615](file:///K:\mouse_ovalbumin_2016\RNA_TBD150541_20160418\00_report\report\report\GO\cellular_component\GO_0005615.html#S0001-S0002) | 9.577006e-17 | extracellular space | 37 | extracellular space |
| 2 | [GO:0005576](file:///K:\mouse_ovalbumin_2016\RNA_TBD150541_20160418\00_report\report\report\GO\cellular_component\GO_0005576.html#S0001-S0002) | 2.32261e-12 | cellular_component | 60 | extracellular region |
| 3 | [GO:0044421](file:///K:\mouse_ovalbumin_2016\RNA_TBD150541_20160418\00_report\report\report\GO\cellular_component\GO_0044421.html#S0001-S0002) | 2.429519e-10 | extracellular region | 51 | extracellular region part |
| 4 | [GO:0000922](file:///K:\mouse_ovalbumin_2016\RNA_TBD150541_20160418\00_report\report\report\GO\cellular_component\GO_0000922.html#S0001-S0002) | 0.0001371716 | intracellular organelle part | 6 | spindle pole |
| 5 | [GO:0009897](file:///K:\mouse_ovalbumin_2016\RNA_TBD150541_20160418\00_report\report\report\GO\cellular_component\GO_0009897.html#S0001-S0002) | 0.0003782244 | cell part | 8 | external side of plasma membrane |
